# Nano-topography Enhances Communication in Neural Cells Networks

**DOI:** 10.1038/s41598-017-09741-w

**Published:** 2017-08-29

**Authors:** V. Onesto, L. Cancedda, M. L. Coluccio, M. Nanni, M. Pesce, N. Malara, M. Cesarelli, E. Di Fabrizio, F. Amato, F. Gentile

**Affiliations:** 1 Department of Experimental and Clinical Medicine, University of Magna Graecia, 88100 Catanzaro, Italy; 2 Istituto Italiano di Tecnologia, Via Morego 30, 16163 Genova, Italy; 3 Department of Electrical Engineering and Information Technology, University of Naples, 80125 Naples, Italy; 4 King Abdullah University of Science and Technology, Thuwal, 23955-6900 Saudi Arabia

## Abstract

Neural cells are the smallest building blocks of the central and peripheral nervous systems. Information in neural networks and cell-substrate interactions have been heretofore studied separately. Understanding whether surface nano-topography can direct nerve cells assembly into computational efficient networks may provide new tools and criteria for tissue engineering and regenerative medicine. In this work, we used information theory approaches and functional multi calcium imaging (fMCI) techniques to examine how information flows in neural networks cultured on surfaces with controlled topography. We found that substrate roughness *S*_*a*_ affects networks topology. In the low nano-meter range, *S*_*a*_ = 0–30 *nm*, information increases with *S*_*a*_. Moreover, we found that energy density of a network of cells correlates to the topology of that network. This reinforces the view that information, energy and surface nano-topography are tightly inter-connected and should not be neglected when studying cell-cell interaction in neural tissue repair and regeneration.

## Introduction

Neural cell adhesion and interaction with artificial substrates is of fundamental importance in neural tissue engineering; bio computing; biosensors operations and neural cell based sensors; diagnosis and analysis of neurodegenerative disorders; neural development. Since neural cells in the central and peripheral nervous systems form functional networks where their efficiency depends on network topology, rational design of synthetic neural tissue substrates should attain maximum control over cell assembly and clustering. Properties of neural networks emerge more from the complex interplay of simple constituents in tightly connected graphs and less from the specialization of individual neurons^1–5^.

While the search for tissue engineered materials and designs has been limited for a long time to properties like biocompatibility, biodegradability, porosity, chemical and mechanical properties^6^, recent advances in nanotechnology unearthed the need of understanding the role nano-topography and nano-geometry at the cell surface interface^7, 8^. As the natural extracellular matrix (ECM) provides a natural environment of intricate nanofibers to support cells and present an instructive background to guide their behavior^9^, surfaces with a controlled nano-geometry may represent an analogous of EMC to the adhesion and proliferation of neural cells^8, 10–12^.

The mechanisms of cell-surface interaction and effects thereof have been examined in a number of studies. Exploring a variety of geometries, including anisotropic gratings, islands of carbon nanotubes, ridges and pillars, and randomly rough surfaces, researchers demonstrated that a nano-scale architecture may direct, control and, in some cases, improve neuronal polarity^13^, adhesion^14, 15^, growth^16, 17^, differentiation^18–20^, organization or self-organization into simple to complex networks^21, 22^, electrical signaling^23^. In other reported experiments^24, 25^, some of the authors of the present work demonstrated that the adhesion and proliferation of various cell linages is maximized on surfaces with moderate roughness and large fractal dimension. Recently, the adhesive behaviour of neuroblastoma N2A cells was verified over porous silicon with a fixed^26^ or smoothly variable pore size^27^.

Preliminary analysis on the topological properties of N2A cells networks on nano-structured surfaces was conducted in ref. 28. It was demonstrated that N2A cells on a surface modified at the nano-scale have an increased ability to create patterns in which the nodes of the patterns form highly clustered groups and the elements of the groups are connected by a finite, and generally low, number of steps, in contrast to a nominally flat surface, where neurons are uniformly distributed. Networks with similar characteristics are named small world networks^3–5^. Small world graphs lie between the extremes of order and randomness^4, 29^. Dynamical systems with a small world topology may feature enhanced signal propagation speed and computational capabilities compared to regular grids of the same size. Neural networks with a small world topology are compatible with the free-energy principle introduced by Karl Friston^30^ whereby biological systems, and ultimately the brain apparatus, tend to maintain a state of high order and to maximize the information that the output signal values convey about the input signals values. These findings are consistent with previous reports^31^, which show that the functional and anatomical connectivity among individual neurons exhibits small-world architectures.

Here, we studied the organization and signaling of neural cells on planar surfaces with details over multiple scales. Using conventional wet etching procedures, we produced surfaces with controlled roughness comprised in the 0–33 *nm* interval. We observed that cultured neural networks exhibit topological properties that depend on the nano-topography of the substrate. Large roughness values trigger cell assembly into small world networks. Using functional calcium imaging techniques, computer simulation and mathematical modelling, we demonstrated that, 11 days after seeding, small world networks on rough substrates conduct information from 3 to 4 folds more efficiently compared to random networks on flat surfaces (with an effective roughness *S*_*a*_ ~ 0.6 *nm*). Using an argument based on energy methods and cell-surface interaction analysis we explain the improved ability of cells on rough surfaces to create clusters.

## Results

### Fabrication and characterization of rough Silicon substrates

**Figure 1:**
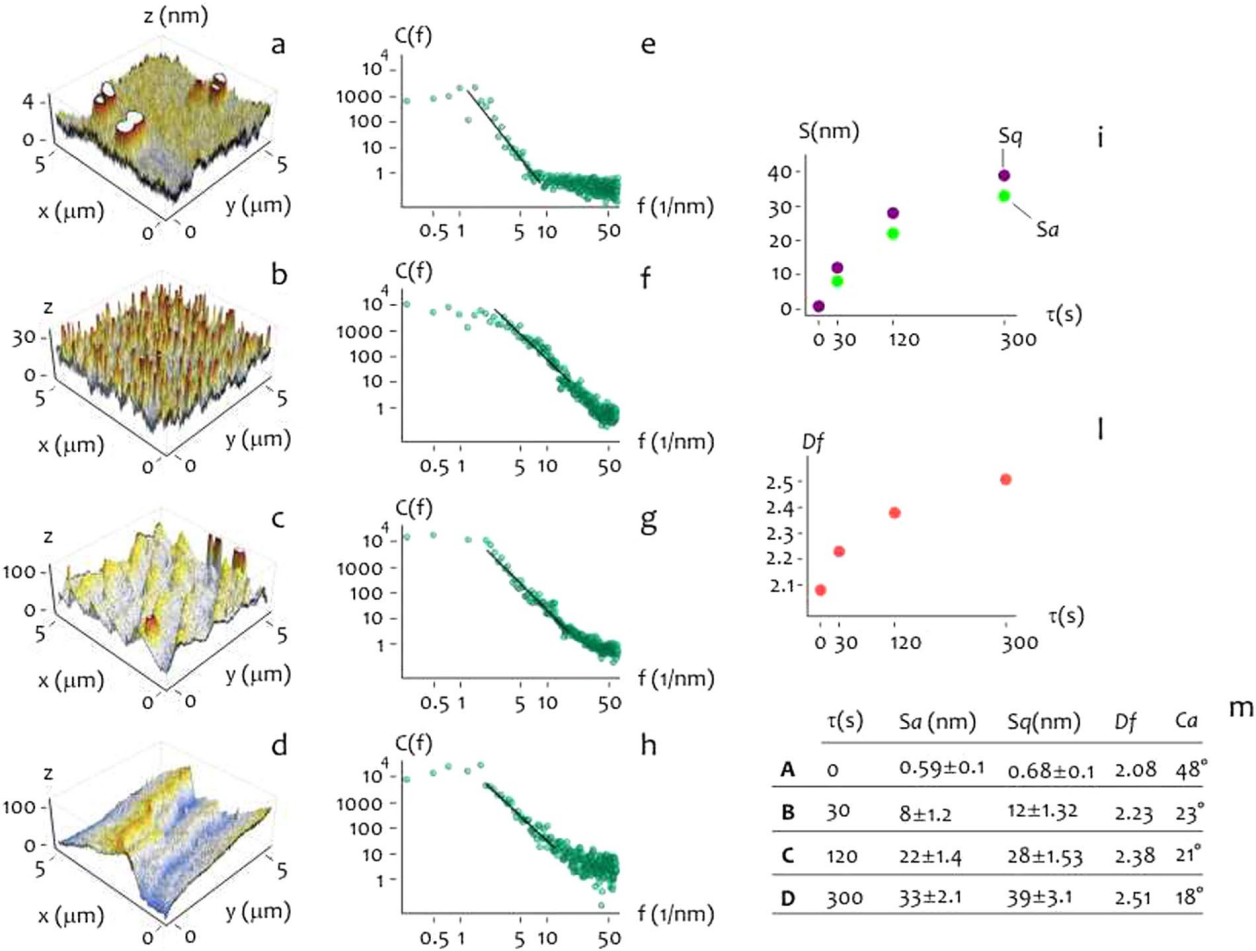
Maintaining silicon surfaces in a corrosive bath for up to 300 s, we obtained rough substrates with varying roughness. AFM images of etched silicon substrates with roughness in the 0.59–33 nm range (**a**–**d**). Corresponding Power Spectrum density functions, which describe the information content of the surfaces over multiple scales (**e**–**h**). From AFM images, average and root mean squared values of roughness were derived (**i**). From Power Spectrum density functions, fractal dimension of surfaces was derived (**l**). The table in the inset recapitulates surface properties for each of the considered time of etching (**m**).

Using conventional wet etching procedures (Methods) we generated rough Silicon substrates with tight control over their topography. Surface nano-topography of samples was examined using standard AFM imaging (Fig. 1). Varying the etching time in a corrosive bath, we obtained samples with an increasing average roughness ranging from ${S}_{a}^{A} \sim 0.6\,nm$ (nominally flat surfaces, Fig. 1a) to ${S}_{a}^{D} \sim 33\,nm$ (extremely rough surfaces, Fig. 1d), with intermediate values of roughness ${S}_{a}^{B} \sim 8\,nm$ (Fig. 1b) and ${S}_{a}^{C} \sim 22\,nm$ (Fig. 1c). Root mean squared roughness ${S}_{q}$ of the same samples displays values that are lightly greater than the arithmetic measure of the roughness profile (Fig. 1i and inset in Fig. 1m). Since roughness parameters ${S}_{a}$ and ${S}_{q}$ reduce all the information in a profile to the deviations from a mean line, they may be insensitive to grossly different spatial and height symmetry features of profiles. In certain conditions, ${S}_{a}$ and ${S}_{q}$ may not be representative of the morphology of a sample unless they are not accompanied by an independent estimate of topography. Here, we use the fractal dimension ${D}_{f}$. The fractal dimension is an index for characterizing patterns by quantifying their complexity as a ratio of the change in detail to the change in scale, therefore, it can be used to describe intimately the topography of small systems^28, 32, 33^. For a bi-dimensional surface the fractal dimension can range from $2$, representing a flat surface (the Euclidian dimension of a surface is $2$) to $3$, representing a severely corrugated surface (the Euclidian dimension of a solid is $3$, in this extreme, one may imagine that a surface explodes into fragments that saturate the space in which it is included). From surface profiles as in Fig. 1a–d we derived the corresponding power spectrum density functions (Fig. 1e–h). A power spectrum (PS) delivers the information content of a surface over multiples scales^32^. In a log-log diagram, a PS exhibits a linear behavior in the central region of the diagram. If the slope of the segment is small (thus the line is horizontal), the PS and the surface are represented over numerous frequencies. Thus fractal dimension and the slope of the PS are correlated. From the measure of $\beta$ one may obtain ${D}_{f}$ as described in the Methods. For the present configuration ${D}_{f}^{A} \sim 2.08$, ${D}_{f}^{B} \sim 2.23$, ${D}_{f}^{C} \sim \,$2.38, ${D}_{f}^{D} \sim 2.51$, and thus in this dimensional range the fractal dimension is proportional to the roughness and steadily increases moving from sample $A$ to sample $D$. High degree of fractality of samples $B-D$, compared to the low fractal dimension of the pristine silicon substrate resembling an ideal Euclidian surface, may be responsible for the increased ability of etched substrates to accelerate cells clustering as commented in the rest of the paper. Contact angle measurements of the samples (Fig. 1m) show that samples are hydrophilic in the considered range of roughness, with contact angle $\theta$ varying from $\theta =48^\circ$ for the flat silicon surface, to $\theta =33^\circ$, $\theta =31^\circ$ $\theta =28^\circ$ for the nano-structured surfaces.

### Cell assemblies in small world networks

**Figure 2:**
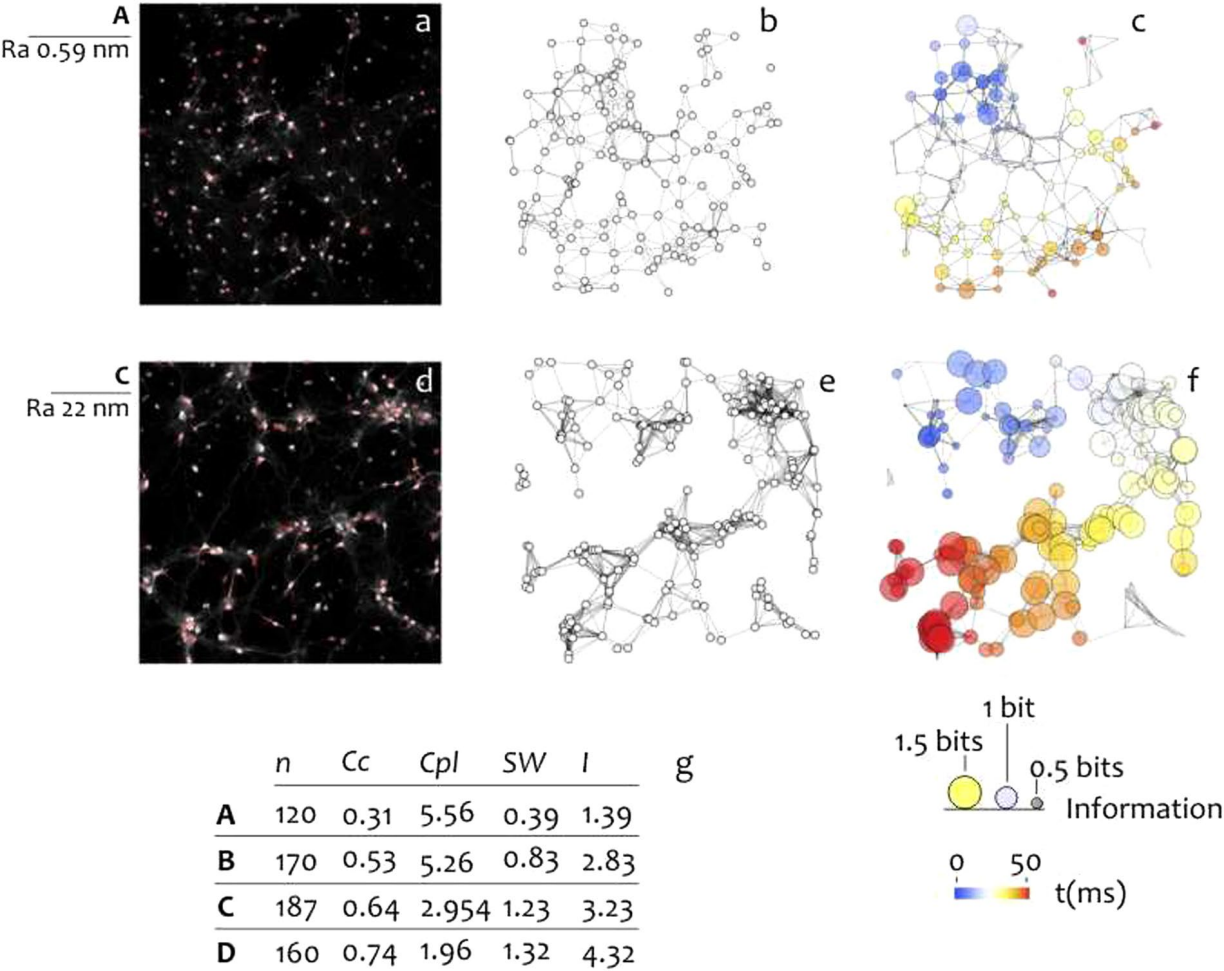
Confocal images of neural cells on flat (**a**) and corrugated (**d**) silicon substrates. Positions of cells nuclei were extracted from confocal images and used to construct cell-graphs (**b**,**e**). Computer simulations were used to derive the Shannon information entropy transported through the networks (**c**,**f**). Results show that information correlates to surface roughness; surface roughness, in turn, boost cell assembly into small world networks (**g**).

**Figure 3:**
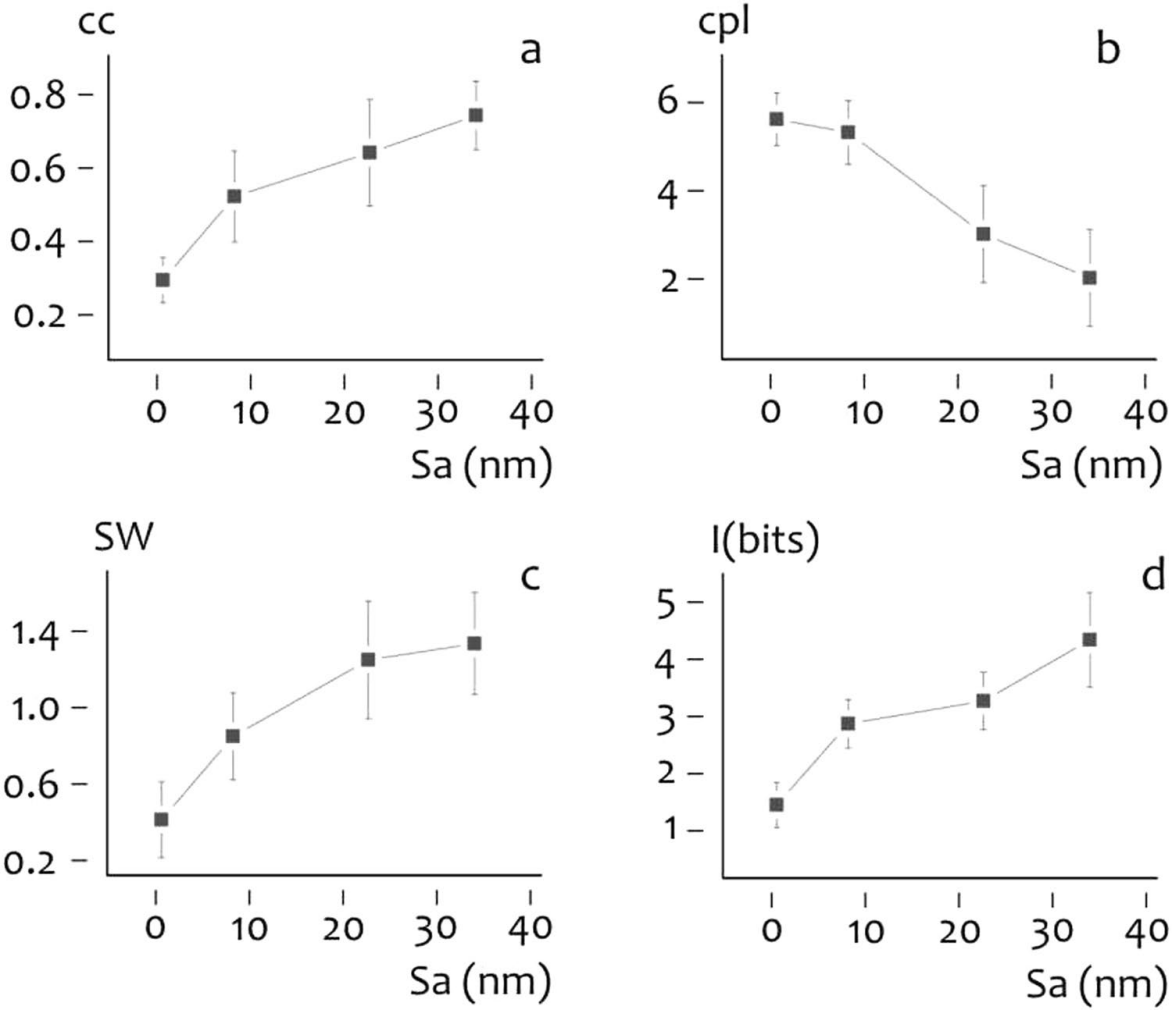
Diagrams summarize clustering coefficient (**a**), characteristic path length (**b**), small-world-ness (**c**), and simulated information in cultured neural networks (**d**) as a function of surface roughness.

In culturing neural cells on the substrates we observed that after $11$ days from seeding cells display different ability to create clusters depending on substrate roughness. Cells adhering within a region of interest (ROI) of $\sim 975\times 750\,\mu {m}^{2}$ were imaged using fluorescent microscopy following the procedure described in the Materials and Methods. For each substrate, more than $30$ ROIs were considered to provide large sample sizes for robust statistical analysis. In Fig. 2 we report fluorescent images of cells adhering over a nominally flat substrate (${S}_{a} \sim 0.6\,nm$, a) in contrast to cells cultured on a corrugated substrate (${S}_{a} \sim 22\,nm$, d). From fluorescent images, the nuclei of the cells were extracted and used to form the networks reported in Fig. 2b (flat Si substrate) and e (rough Si substrate), in which the components of the circuit (the nodes) interact through edges which connect doublets of nodes, and the density of connections is larger if inter-nodal distance is smaller^28, 34^. One may observe that cells are uniformly distributed over the smooth surface; differently, cells over the corrugated profile form aggregates where in each aggregate cell to cell distance is low and the separation between aggregates is high. One way to examine the topology of a group of elements in a plane is using variables like the clustering coefficient and the characteristic path length. The clustering coefficient ($Cc$) describes the propensity of the nodes of a graph to form few groups in which the elements of the groups are inter-connected by the an elevated number of edges^34–36^. The characteristic path length ($Cpl$) indicates the number of passages that on average separates two nodes randomly picked in a network^36^. $Cc$ is comprised between $0$ and $1$, $Cpl$ is generally greater than $1$ (Materials and Methods). $Cc$ and $Cpl$ are used to describe and assess the efficiency of complex systems and dynamical systems^3, 5^. Networks with high $Cc$ and low $Cpl$ are named small world networks. Small world networks typically feature over-abundance of hubs with a high number of connections. Thus networks with a small world architecture may mediate information between nodes of the network and function more efficiently than equivalent random, periodic or regular graphs^3, 5^. More precise definition of small world networks is contained in the Methods and in the Supporting Information File 1. The degree of small-world-ness of a network is indicated by the sole coefficient ${\rm{SW}}$. Small world networks have ${\rm{SW}} > 1$ (Methods). In the considered range of roughness we found that cultured neural networks exhibit (i) increasing $Cc$, (ii) decreasing $Cpl$ and consequently (iii) increasing ${\rm{SW}}$ values for increasing roughness (Table in the inset of Fig. 2g and Fig. 3). ${\rm{SW}}$ index smoothly transitions from $\sim 0.4$ for the ${S}_{a} \sim 0.6\,nm$ substrate to $\sim 1.3$ for the ${S}_{a} \sim 33\,nm$ substrate. While cells on flat substrates present no small-world-ness attributes (${\rm{SW}} \sim 0.4$), moderately rough surfaces (${S}_{a} > 22\,nm$) boost the organization of nerve cells into small world networks (${\rm{SW}} > 1$). Crossover between the absence and the presence of a small world architecture of neural networks is observed in the intermediate nanometer range. In the Table in Fig. 2g the number $n$ of adhering cell is reported as a function of sample preparation. $n$ and thus cell density vary in narrow intervals moving from sample $A$ to sample $D$. This would suggest that networking of neural cells on a substrate is not influenced by cell density and is regulated by the sole substrate topography. Cell images as in Fig. 2a,d are examples extracted from larger data sets. Full list of wiring diagrams of cells for all considered substrate preparations is reported in a separate Supporting Information File 2.

### Simulating information flow in cultured neural networks

Reported results show that neurons on rough substrates form clustered networks in opposition to neurons on flat surfaces. For these configurations, neurons are connected by a finite and general low number of steps and information in the grid may be transported more efficiently. Computer simulations (Methods) confirm this hypothesis. We reproduced artificial neural networks from confocal images of the cells for all the considered substrates (Fig. 2b,e). Then, elements of the networks were excited with a variable function of time. Upon excitation, spikes propagate in cascade in the grid. Time spikes are grouped in sets of words, in which a word is an array of on (presence of a spike)/off (absence of a spike) events in a binary representation (Supporting Information Figure 3.4). On sorting words in order of decreasing occurrence in the train, we derived the associated entropy using equation (Supporting Information S2.3) and the Methods described in the Supporting Information File 3. We repeated this procedure in response to an uncorrelated (Supporting Information Figure 3.3a) and time locked (Supporting Information Figure 3.3b) signal of time. From the difference of entropies, we derived the information transmitted all over the nodes of the grid for cells cultured on flat (Fig. 2c) and etched (Fig. 2f) surfaces. We found that the simulated information steadily increases moving from random to small world graphs and thus with substrate roughness (Fig. 3d). We measured an augment of information from $I \sim 1.4$ bits for the ${S}_{a} \sim 0.6\,nm$ substrate to $I \sim 4.3$ bits for the ${S}_{a} \sim 33\,nm$ substrate, with a $\sim 3$ fold overall increase.

### Ensemble Dynamics of Spontaneous Activity

**Figure 4:**
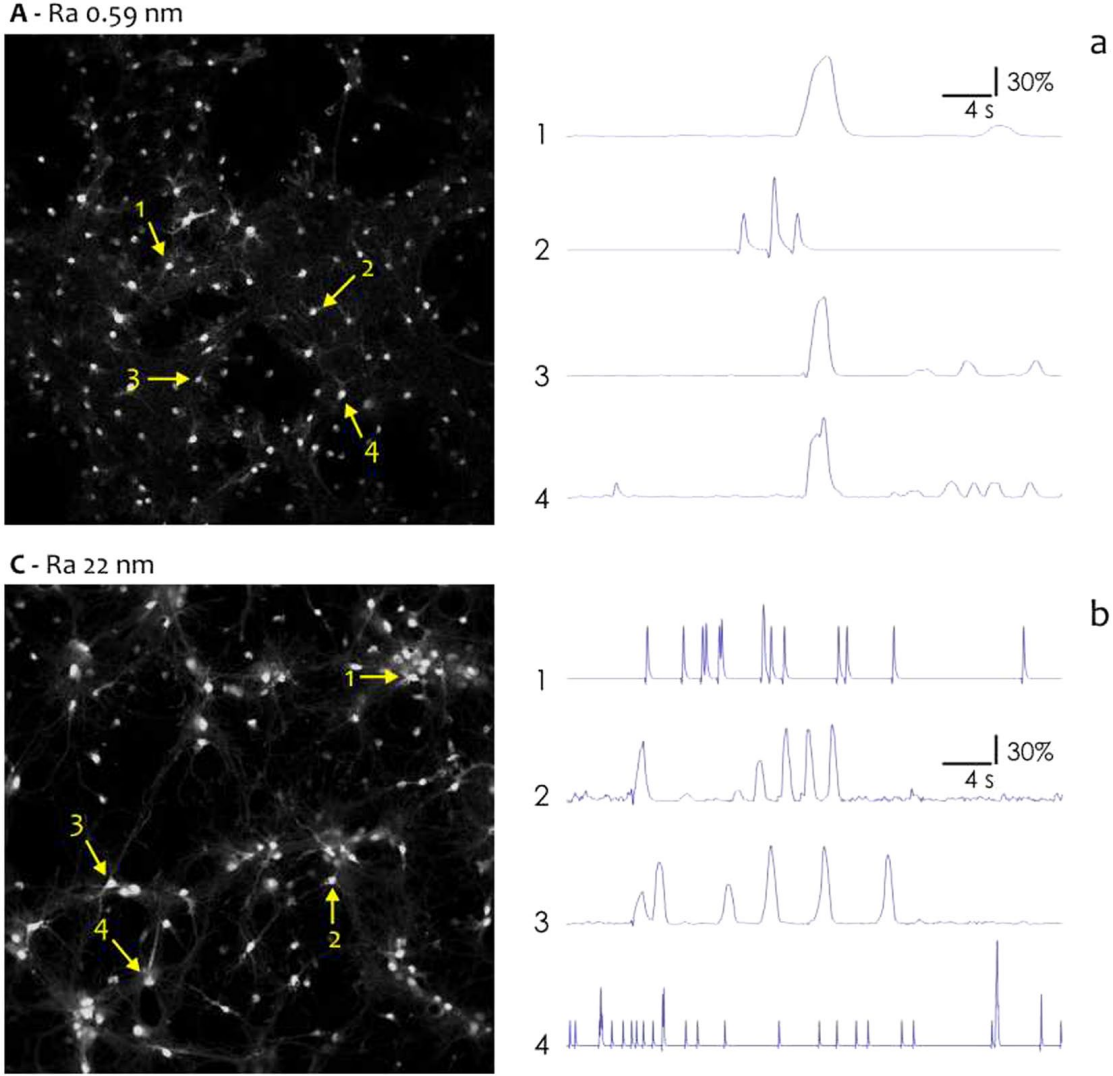
Functional multi calcium imaging (fMCI) techniques were used to obtain the spontaneous activity profile of individual neurons in cultured neural networks on flat (**a**) and corrugated (**b**) surfaces.

**Figure 5:**
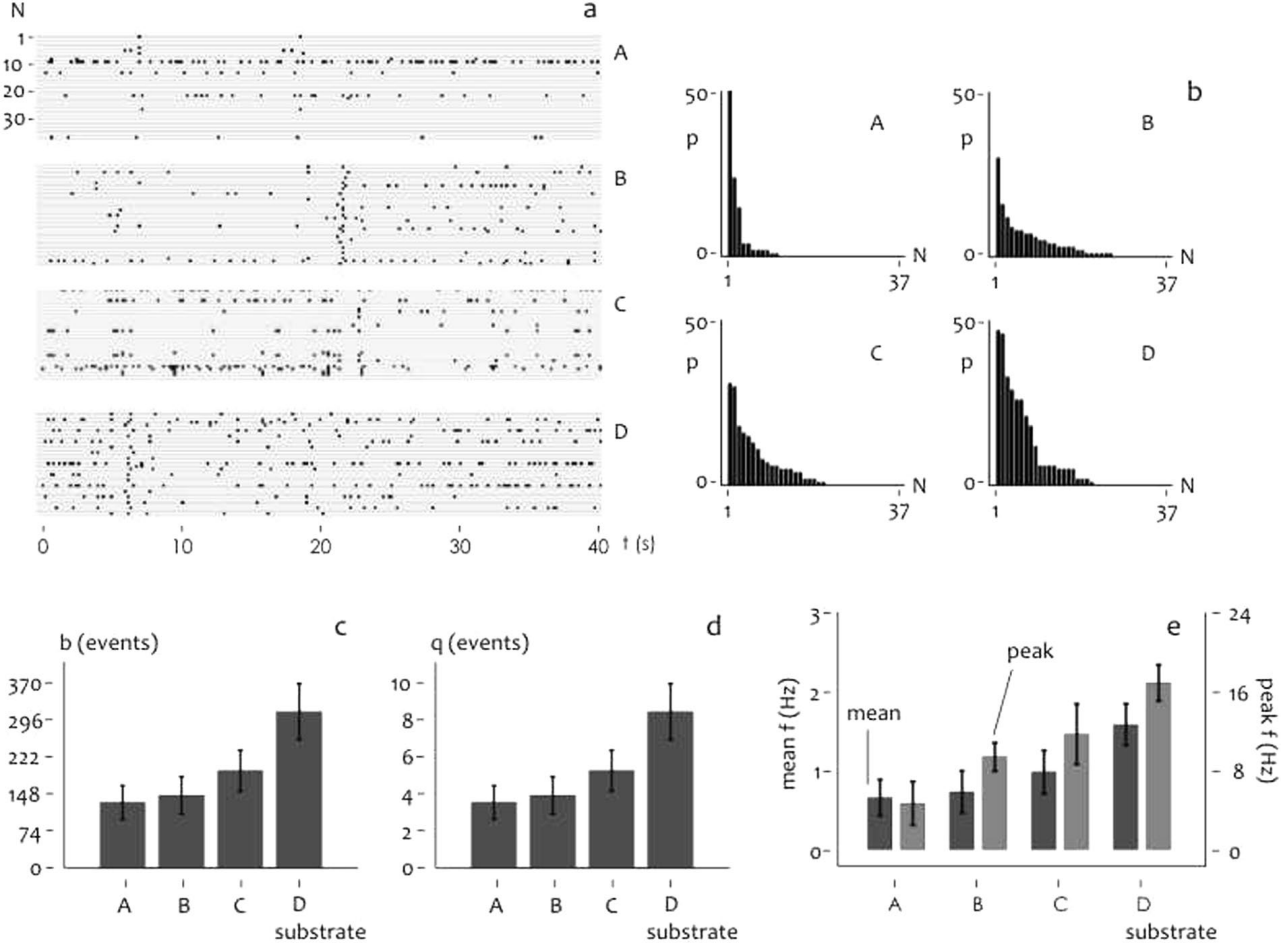
Raster plots describe spontaneous activity of 37 neurons per each considered surface roughness, in the plots measured neuron activity is reported as a dot in a binary representation (**a**). Histograms of neuronal activity reveal that more than 50% of neural population is involved in network activity for corrugated surfaces (B–D), in contrast to flat surfaces (A) where it is registered a limited neuronal activity (**b**). Total (**c**) and averaged (**d**) number of spikes measured in cultured neural networks as a function of substrate preparation. Mean and peak firing rate measured in cultured neural networks as a function of substrate preparation (**e**).

We used high-speed fMCI to examine the dynamics of spontaneous firing activity of neuron populations. The spatio-temporal pattern of spontaneous network activity was reconstructed with the millisecond resolution from $37$ neurons for each substrate topography. Figure 4 reports confocal images and associated neural activity for neurons over smooth (a) and moderately corrugated ${{\rm{S}}}_{{\rm{a}}} \sim 22\,\text{nm}$ substrates (b). In cultured neural networks $37$ neurons were randomly selected for fMCI recordings. Of $37$ neurons, a reduced sample of $4$ neurons is reported in Fig. 4 for sake of clarity. Spikes of spontaneously active neurons were determined as somatic ${{\rm{C}}}_{a}^{2+}$ transients as described in the Methods. Spikes were registered throughout a time interval of $40$ s and reported in the right hand panel of Fig. 4a,b as variation respect to the baseline. Closely spaced spikes are observed in small world networks over corrugated surfaces (Fig. 4b) suggesting that neural small world networks are topologically biased to enhance local connectivity. Data are summarized in Fig. 5. Network burst profiles are reported in Fig. 5a for different substrate preparations from sample ${\rm{A}}$ with ${{\rm{S}}}_{{\rm{a}}} \sim 0.6\,\mathrm{nm}$ to sample ${\rm{D}}$ with ${{\rm{S}}}_{{\rm{a}}} \sim 33\,\mathrm{nm}$. Neuron spiking events are reported in a matrix plot as a function of time for all considered neurons in the network. Corresponding spike histograms are shown in Fig. 5b as a function of neuron index. Within a given $40$ s period, on average, only a small number of neurons ($24 \%$ of the total cells) were active on flat surfaces, whereas on rough surfaces the network exhibited more events that involved up to about $50 \%$ neurons (Fig. 5b). Both histograms and bursts time representation reveal that neural activity increases with surface roughness. To quantify the activity level, we introduce the scalar measure $b$, which is the summation of observed action potentials over all neurons in a time interval, and $q$, which is $b$ averaged over neurons in the network. Both $b$ (Fig. 5c) and *q* (Fig. 5d) increase with surface roughness. In particular, *q* transitions from $q=3.48\pm 0.18$ spikes/neuron for S_a_ ~ 0.6 nm to *q* = 8.35 ± 0.41 spikes/neuron for S_a_ ~ 33 nm. Thus ratio of density of spikes measured on flat surfaces to that measured on rough surfaces is ~2.4, this proportion is consistent with values predicted by information theory simulations reported in paragraph 3.3. The values of *b* and *q* correlated positively with S_a_ (*r* = 0.21, *P* < 0.01). For each of the considered substrate, mean and peak firing rate of neurons in the networks were extracted from the raster graphs of Fig. 5a as (i) average number of events registered on a network to the predetermined time interval and (ii) mean ratio of 10 most closely spaced events in a grid to their time distance (Fig. 5e). Neurons firing rate *f* increases with roughness ranging from ~6 Hz for S_a_ ~ 33 nm to ~16 Hz for S_a_ ~ 33 nm. These data imply that excitatory neurons are specifically wired to ensure enhanced neural activity in small world networks over corrugated surfaces.

## Discussion

### Information in planar neural cultures correlates to network topology

Using computer simulations and fMCI techniques we found that the information in planar neural networks increases with the small-world-ness of the networks. Network topology, in turn, is influenced by roughness of the substrate. Thus, one can potentially control the organization of nerve cells into computational efficient networks by modulating substrate nano-topography. In the considered range of roughness, small-world-ness SW of neural networks varies with S_a_ and the SW(S_a_) response recalls the characteristic response of a first order system (Fig. 3c). ${\rm{SW}}$ asymptotically approaches a steady-state value that for the present configuration is ${{\rm{SW}}}_{{\rm{ss}}}=1.32$. We may divide the ${\rm{SW}}$ diagram into two regions: (i) a transient region (${{\rm{S}}}_{{\rm{a}}} \sim 0.6-22\,\text{nm}$) in which ${\rm{SW}}$ varies linearly with ${{\rm{S}}}_{{\rm{a}}}$, (ii) a steady state region (${{\rm{S}}}_{{\rm{a}}} \sim 22-33\,\mathrm{nm}$) in which the system is assumed to have reached its final value ${{\rm{SW}}}_{{\rm{ss}}}$. For ${{\rm{S}}}_{{\rm{a}}} \sim 22\,\text{nm}$, ${\rm{SW}}=1.23$ is within 10% of its final value, thus we may choose ${{\rm{S}}}_{{\rm{a}}} \sim 22\,\text{nm}$ as the boundary between the transient and steady-state responses. Sensitivity analysis shows that network topology is affected largely by surface roughness when S_a_ is comprised in the first transient regime (${{\rm{S}}}_{{\rm{a}}} \sim 0.6-22\,\text{nm}$). For larger values of ${{\rm{S}}}_{{\rm{a}}} > 22\,\mathrm{nm}$, ${\rm{SW}}$ varies negligibly with ${{\rm{S}}}_{{\rm{a}}}$. Remarkably, the observed length scale $\sim 20\,\mathrm{nm}$ for which neurons optimize network topology, coincides with the extension of neurons’ filipodia and lamellipodia during neuronal differentiation^37^. This suggests that one can attain maximum control over network architecture by tailoring surface nano-topography in the low nanometer range. These findings are in line with previously reported experiments, where it was observed that moderately rough surfaces^25^ enhance adhesion and proliferation of different cell linages. In^28^, some of the authors of the present work demonstrated that N2A cells assembly and organization is influenced by the porous architecture of mesoporous silicon surfaces. For the present configuration, we found that the information and overall cell activity of cultured neural networks varies with network topology and thus surface roughness. Information transported in the grid and normalized action potentials release events may be increased from 3 to 4 folds moving from random graphs on flat surfaces to small world networks on etched substrates. Described results reinforce the view that, in a network, information flow, topology of the network and surface topography are densely correlated. Adjusting roughness, one can modulate the amount of information transported through the grid.

### Energy landscape of small world networks

**Figure 6:**
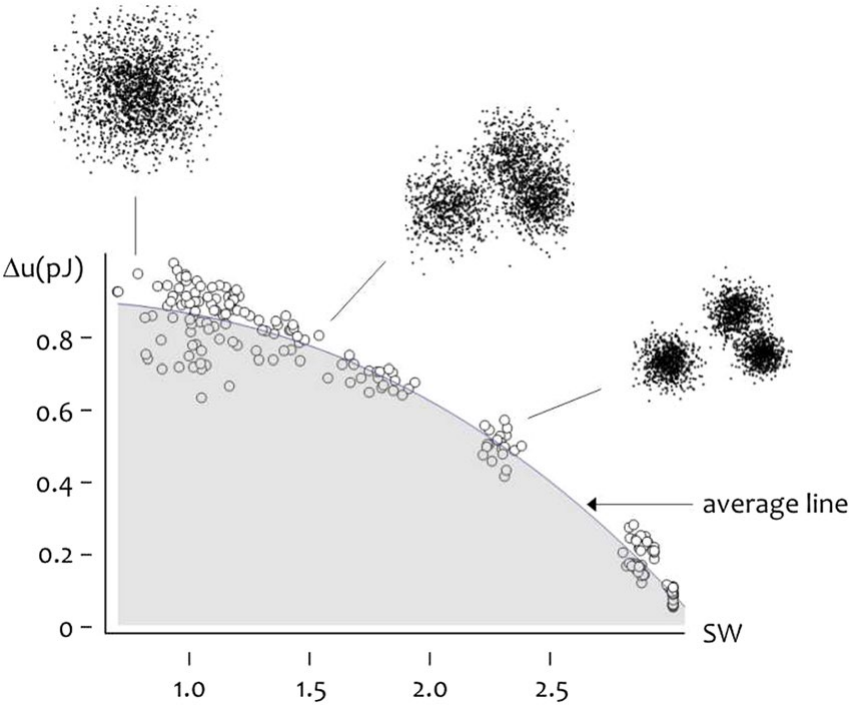
Calculated energy landscape of neural networks as a function of small-world-ness.

Figure 6 describes the density of energy change Δ*u* as a function of small-world-ness associated to a system of 200 cells on a planar surface. Energy density was derived by summing the potential energy of interaction between cells over all the possible cell pairs in a network (Methods and Supporting Information File 4). A great many of randomly generated networks exhibit varying topological properties and SW coefficient that ranges from ~0.7 (random graphs) to ~2.9 (small world graphs). The energy landscape in the diagram associates each conformation of the system to its energy levels. Best fit of numerical data is given by the quadratic form $0.78+0.189\,{{\rm{SW}}}^{2}-\,0.147\,{{\rm{SW}}}^{2}$, thus the total free energy smoothly decreases from $0.9\,\mathrm{pJ}/{\rm{bond}}$ for ${\rm{SW}}=0.7$ to $0.8\,\mathrm{pJ}/{\rm{bond}}$ for ${\rm{SW}}=1.5$, to $0.1\,\mathrm{pJ}/{\rm{bond}}$ for ${\rm{SW}}=2.9$. Since physical systems evolve to maintain their free energy to a minimum, Fig. 6 indicates that, while cells can theoretically exist in a nearly infinite number of conformations along their energy landscape, in reality cells would tend to assemble into clustered groups that exhibit lower energy levels. In this interpretation, few groups of highly clustered cells (elevated SW values) represent the natural conformation of bi dimensional systems of neural cells. Remarkably, this conformation is encountered on corrugated nano-scale systems instead that on smooth surfaces. Thus, rough surfaces prompt cells to assembling into small world networks that (i) minimize energy and (ii) maximize information density:

1.1 $${\rm{rough}}\,{\rm{surfaces}}\to {\rm{SW}}\,{\rm{neural}}\,{\rm{networks}},$$

1.2 $${\rm{low}}\,{\rm{energy}}\,{\rm{density}}\leftrightarrow {\rm{high}}\,{\rm{information}}\,{\rm{flows}}$$

Figure 6 uses energy variables to recast the organization of cells on a substrate problem in energetic terms and along with relations (1) states the equivalence between energy and information in systems of neural cells. The different ability of rough surfaces to boost the spontaneous organization of cells into clusters in contrast to smooth surfaces is explained below.

### Roughness breaks equilibrium and triggers cells clustering

**Figure 7:**
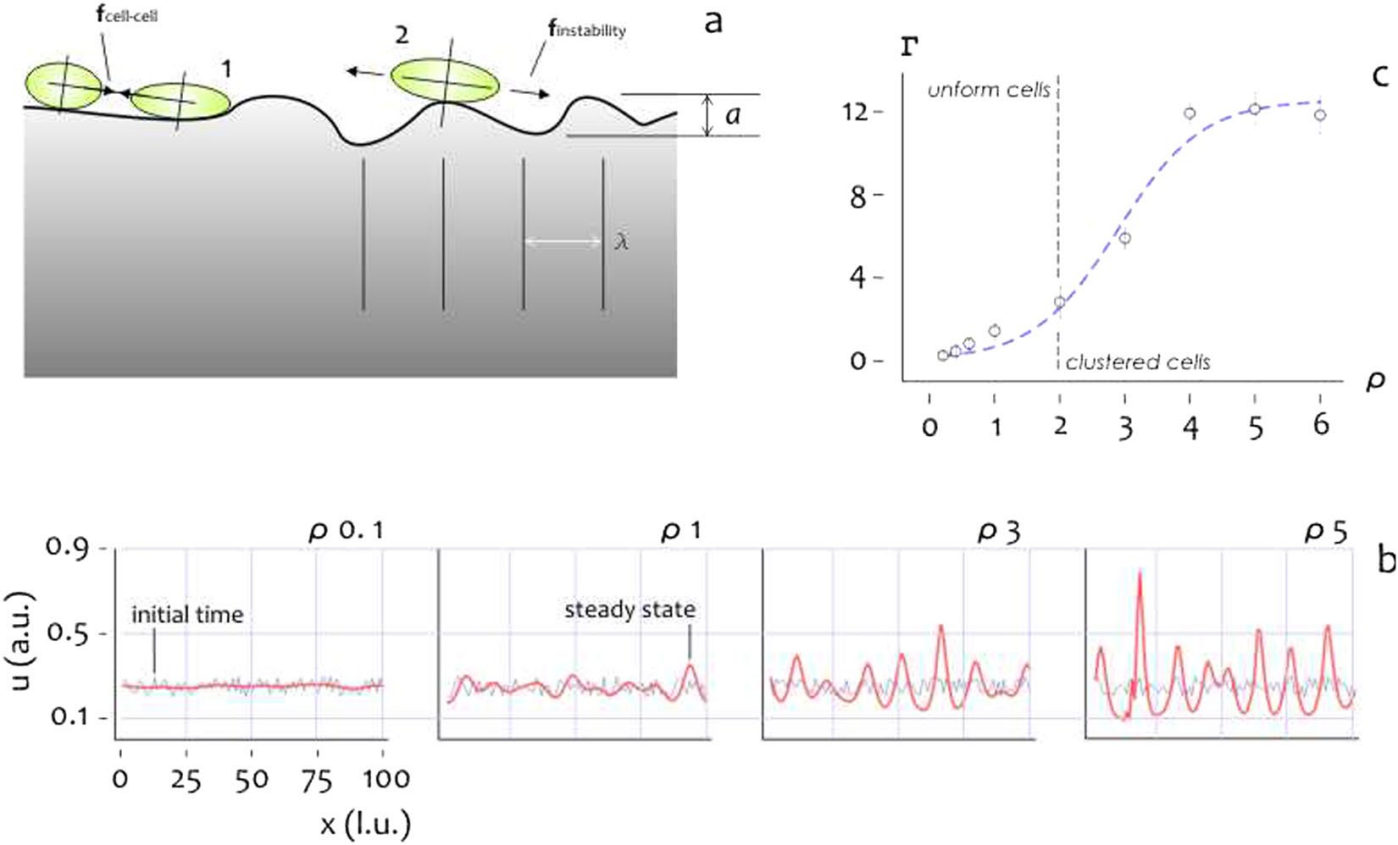
Cells on a corrugated surface experience cell-cell interactions and an instability force generated by surface roughness, whereby cells, at the early time of adhesion, move and deform their membrane to adapt to surface profile (**a**). Initial and steady state solution of cell density for different values of surface instability force relative to cell-cell adhesion force (**b**). Cells form isolated groups of cells if the surface instability force relative to cell-cell adhesion force is greater than one (**c**).

Cells motility on a surface is determined by cell-cell (1) and cell-substrate (2) interactions (Fig. 7a). To describe the time evolution of cell density, we use a revised version of the continuum model described in ref. 38, in which cell density obeys to a partial differential equation of time and space (Methods and Supporting Information File 5). For numerical convenience, we consider Equation 4 in a mono-dimensional domain described by the sole spatial *x* coordinate. In the equation, cell density is a function of cell-cell adhesion forces, of intensity $\xi$, and of the forces induced by substrate instability, whereby cells initially seeded on a surface explore near sites to conform to the surface profile and minimize energy^39^. Force induced by substrate instability is proportional to the parameter γ. Thus ${\varrho }=\gamma /\xi$ is the relative intensity of the substrate instability force to the cell-cell adhesion force. Steady state solution of cell density is reported in Fig. 7b for ${\varrho }$ ranging from $0$ to $6$. We observe that while for small values of ${\varrho }$ (${\varrho } < 2$) cells density is uniform, when ${\varrho } > 2$ cells cluster together to form isolated peaks. Diagram in Fig. 7c describes asymptotic cell behavior as a function of ${\varrho }$. Value ${\rm{\Gamma }}$ in the ordinates is the average ratio of maximum (peaks) to minimum (valleys) values of cell density at regime, thus large ${\rm{\Gamma }}$’s are suggestive of cells clustering. Remarkably, two regions are distinguishable in the diagram. When cell-cell adhesion forces dominate over substrate perturbation forces (${\varrho } < 2$) we have isolated cells in the domain, when substrate perturbation forces dominate over cell-cell adhesion forces (${\varrho } > 2$) cells create clusters. Similarly to what happens for undercooled liquids^40^, cells may maintain a state of unstable equilibrium and remain uniformly distributed on a surface contradicting the energy landscape in Fig. 6. Surface roughness, lumped here in the sole γ parameter, breaks equilibrium and drives cells clustering. In^39^, it was calculated that early energy variation for a cell is in the order of $U \sim 1\,\mathrm{fJ}$ on a substrate with roughness ${S}_{a} \sim 20\,nm$ for an initial surface energy ${\gamma }_{c} \sim \,1\,{\rm{\mu }}J/{m}^{2}$. Thus, force associated to a similar energy variation is $F=\partial U/\partial x \sim 100\,pN$, that is in the same dimensional range of cell-cell adhesion forces^41, 42^. Data presented in Fig. 7 seem to support the notion of rationally designing surfaces by tailoring nano-topography to modulate cell clustering.

### On the generality of the results and applications

The findings of the work are descriptive of nerve cells especially pertaining information propagation, the profiling of time spikes and simulation of information flow in networks using information theory variables. Nevertheless, some other findings are general in nature. Cell clustering, the formation of small world networks and energy minimization in those networks are universal mechanisms that can be applied to the large majority of cell types. This is due to the fact that cell clustering is activated by cell-cell and cell-surface interactions that are not exclusive of nerve cells. In deriving free energy landscapes of small world networks, we used a chemical potential that describes the chemical interaction energy between cells due to specific and non-specific adhesion forces. In determining the time evolution of cell population on a substrate, we introduced a term (${\varrho }$) that reflects the relative intensity of the substrate instability forces to the cell-cell adhesion forces. Thus one can use these methods to describe the behavior of diverse cell types using precise combinations of the chemical potential of interaction and ${\varrho }$.

As regarding the *equivalence* between cell organization and networking, information and energy in a grid (Equations 1.1–2): this may represent a tool for addressing the problem of cell growth and assembling under different perspectives in a variety of disciplines, including regenerative medicine, tissue engineering, the diagnosis or therapy of neurodegenerative disorders. Assuming that the main drivers for neural cells organization in the plane are

(i) *Energy optimization*, whereby cells create networks which minimize thier free energy;
(ii) *Neural morphogenesis*, whereby cell positioning on the substrate is determined by the morphology and biology of neurons, and from cell-cell and cell-surface interactions;
(iii) *Information propagation*, whereby nerve cells form networks which maximize information in a grid;

In establishing the correspondence between the energy, biology and information criteria, we suggest that these criteria can be used interchangeably. That is to say that the same problem can be addressed from different sides. Thus, making experiments or studies in one of those domains, one would obtain the information necessary to design strategies in the remaining domains. Similarly in concept to the Fourier transform that, on transforming a signal from the time to the frequency domain, allows to solve a problem in the more convenient conditions and then to transfer the solution to the original domain. Possible applications of this method may be (but not exclusively) in (i) neuro-tissue engineering and scaffold design, (ii) the analysis and diagnosis of neurodegenerative disorders; (iii) bio-computing. In the first case (i): on determining the surface energy density of a substrate, one can anticipate and predict cell-organization and clustering on that substrate. In the second case (ii): scientists may correlate the lack of information in a network to an anomalous production/depletion of specific neurotransmitters or biomarkers in that network. If detected in the body, similar biomarkers would reveal the occurrence and progression of neurodegenerative diseases. In the third case (iii): equivalence between information, energy and biology, may help in the design of scaffolds and 2 to 3*D* substrates that, in optimizing cell clustering, maximize the computational capabilities of biological systems.

## Conclusions

We cultured neural networks on rough surfaces. On varying the roughness of the surfaces over a significant range, we found that the topological properties of the networks show a very high sensitivity to surface topography. Cells on corrugated surfaces (${S}_{a} > 22\,nm$) exhibit small world attributes, whereas cells on nominally flat surfaces (${S}_{a} < 10\,nm$) are uniformly distributed without clustering effects. Using computer simulations and fluorescent multi calcium imaging techniques, we found that small world networks on corrugated surfaces are computational efficient. Information in small world networks is enhanced up to 4 times compared to information exchanged in unstructured networks over flat surfaces. Using energy methods, we found that cells in small world groups minimize their energy, thus systems of cells would spontaneously evolve into clustered geometries if they surpass an initial energy barrier. Nano-scale structure of the surface provides the energy necessary to overcome this barrier. Presented results show that nano-topography, information and energy are intimately correlated, and should be jointly considered in the rational design of neural tissue substrates.

## Materials and Methods

P-doped (111) wafers with 5–10 Ohm/cm resistivity were used as substrates (Si-Mat, Kaufering, Germany). De-ionized (D.I.) water (Milli-Q Direct 3, Millipore) was used for all experiments. Potassium hydroxide (KOH), ethanol, methanol were purchased from Sigma Aldrich (Milan, Italy). All culture media and reagents were from Invitrogen (Milano, Italy), unless otherwise specified. All chemicals, unless mentioned otherwise, were of analytical grade and were used as received. Experiments were performed on day 11 *in vitro*.

### Realizing rough silicon surfaces

Planar Silicon surfaces with a roughness *S*_*a*_ comprised in the $0-30\,nm$ range were fabricated using the methods described in ref. 24. Smooth silicon surfaces with a residual roughness of about 5 Angstroms were used as a control. Silicon wafers were etched in diluted potassium hydroxide solutions (${\rm{KOH}}:\mathrm{DI}\,\mathrm{water}=1:4$) to obtain corrugated profiles. Varying the etching time from 0 to 300 s, we obtained surfaces with an average roughness ${S}_{a}$ ranging from $\sim 0.6\,nm$ (nominally flat surfaces) to $\sim 33\,nm$ (Fig. 1l–m).

### AFM sample characterization

Atomic force microscopy (integrated Raman AFM system, Alpha 300 RA, Witec) was used for sample characterization. All the measurements were performed in a dry environment in intermittent contact mode over a sampling area of 5 × 5 μm^2^. Room temperature was hold fixed for all the acquisitions. Ultra-sharp Si probes with a nominal tip radius less than 5 nm were used for achieve high resolution. Multiple measurements were done in different scan directions to avoid artefacts. At least four images in height mode (trace and retrace) were recorded per each sample. The images had a resolution of 512 × 512 points and were acquired at a scanning rate of 1 Hz. Images were processed using either flattening or plane fit according to the relief characteristics, with the minimal polynomial order needed. The characteristic average surface roughness ${{\rm{S}}}_{a}$ was thus de-convolved for each substrate. Fast Fourier transform (FFT) algorithms were used for data processing and fractal extraction of the characteristic dimension of the samples surface.

The etching of the structure is extremely homogeneous compared to the whole surface. Silicon chips are square surfaces with an edge of approximately $10\,mm$. Chips are immersed in a corrosive $KOH$ bath that guarantees uniformity of etching over the entire surface. AFM measurements on randomly picked regions on the chip surface reveal that the measured profile is regular over the entire surface. Moreover, individual AFM measurements reveal that roughness is periodic and regular within the windows of measurement. Considering that the characteristic wavelength of etched surface is smaller than $100\,nm$ for all considered cases, lateral resolution of the structures is about $100\,mm/{10}^{7}\,nm={10}^{-5}$ times the maximum length of the chips. Thus, rough surfaces are smooth and homogeneous at the macroscale. Even considering not the entire chip but the region of interest (ROI) within which confocal analyses were performed, that is about 1 mm in length, the ratio of the profile wavelength to the ROI size is still extremely small: $100\,mm/{10}^{6}\,nm={10}^{-4}$. Thus, while nanoscale architecture of the substrate influences cell behavior and clustering, cells distribution would not depend on spatial irregularities of surface nano-structuring, that is uniform over the substrate.

### Deriving the fractal dimension of the surfaces

AFM profiles of the surfaces were processed using the algorithms developed and described in references^32^. We derived the characteristic power density function for each surface (Fig. 1e–h). In a log-log plot, the power spectrum density appears as a line with a slope β. The slope β is related to the Hurst parameters as ${\rm{\beta }}=2(H+1)$. The fractal dimension ${D}_{f}$ of the surface can be equivalently derived as ${D}_{f}=(8-{\rm{\beta }})/2$ or ${D}_{f}=3-H$. The fractal dimension ${D}_{f}$ of a surface ranges from 2 (Euclidean dimension of a flat surface) to 3 (representing an extremely rough surface).

### Surface contact angle measurement

Surface hydrophilicity of the samples was determined by measuring the water contact angle with one drop of about $5\,\mu l$ of DI water using an automatic contact angle meter (KSV CAM 101, KSV Instruments LTD, Helsinki, Finland) at room temperature. Four measurements were performed on each substrate to evaluate the average contact angle at $5\,s$.

### Substrate preparation

Silicon rough substrates ($1\,c{m}^{2}$) were individually placed in 35 mm tissue culture dishes (Corning Incorporated), sterilized by immersion in ethanol, washed twice in ${H}_{2}O$, dried in a laminar flow hood and further sterilized by UV irradiation for $2\,h$. To coat the substrates, Poly-D-lysine (PDL) (Sigma-Aldrich, Milan, Italy) was diluted in sterile ${H}_{2}O$ to a final concentration of $1\,\mu g/ml$. Substrates were let in the PDL solution overnight in a cell culture incubator (37 °C, $5 \% \,C{O}_{2}$, 5% humidity).

### Primary neuronal cultures on the substrates

Whole brains were extracted from C57B/L6 mouse embryos at day 18 (E18). All procedures were carried out in accordance with the guidelines established by the European Communities Council (Directive of November 24th, 1986) and approved by the National Council on Health and Animal Care (authorization ID 227, prot. 4127, 25th March 2008). Pregnant females were deeply anesthetized with $C{O}_{2}$ and decapitated. Embryos were removed and brains were placed in cold Hank’s Balanced Salts solution (HBSS). After removal of the meninges, the hippocampus was carefully dissected, incubated with 0.125% trypsin for 15 min at 37 °C and mechanically dissociated. Neurons were plated on a PDL coated surface in complete cell-culture medium, supplemented with 10% fetal bovine serum (FBS, Invitrogen), 5% penicillin G (100 U/ml) and streptomycin sulfate ($100\,{\rm{mg}}/{\rm{ml}}$) (Invitrogen). Neurons were incubated for 18 days *in vitro* (DIV) at 37 °C in a humidified 5% $C{O}_{2}/air$ atmosphere with an initial density of 10^5^ cells/ml. Neurons were plated at the same density on PDL-coated smooth silicon substrates serving as a control. Cells were sub-confluent throughout the duration of the experiment.

### Sample preparation for fluorescence imaging

After incubation, cell culture medium was removed and the cells were washed twice in PBS and fixed with 4% PFA (paraformaldehyde) and were incubated for 30 min at room temperature (RT). The cells were washed twice PBS and made permeable with 0.05% triton (Invitrogen) for 5 min at RT. All cells fixed and made permeable were stained with $100\,\mu l$ DAPI (40, 6-Diamidino-2-phenylindole, Sigma Aldrich) solution for 10 min at 4 °C at dark. Finally, the DAPI solution was removed and each sample was washed with PBS.

### Imaging adhering cells on the substrates

An inverted Leica TCS-SP2® laser scanning confocal microscopy system was used to image cells adhering on the substrates. All measurements were performed using a ArUv laser. The pinhole ($80\,\mu m$) and laser power (80% power) were maintained throughout each experiment. Confocal images of blue (DAPI) fluorescence were collected using a 405 nm excitation line and a 10° dry objective. For each substrate, a large number of images was taken for statistical analysis. Each image was acquired over a region of interest of $975\times 750\,\mu {m}^{2}$, and averaged over 4 lines and 10 frames to improve quality and reduce noise. Images were digitalized into 1=280 × 960 pixels.

### Network analysis of neural cells

Confocal images of cultured neural networks were processed to extract the topological parameters of the networks. Using algorithms described in^28^ and recapitulated in a separate Supporting Information File 1, the average cluster coefficient ($Cc$) and characteristic path length ($Cpl$) of the networks were derived as a function of surface roughness. The core of the algorithms is the Waxman model, which makes a decision on whether two nodes in a grid $(u,v)$ are connected or not^34^. The model makes use of a probability function $P(u,v)$ which decays exponentially with the Euclidean distance $d(u,\,v)$ between $u$ and $v$: $P(u,v)\propto {e}^{-d(u,v)/\beta L}$, where $L$ is the largest possible Euclidean distance in the grid and $\beta$ is a parameter. If a randomly generated number between 0 and 1 is smaller than *P*, then nodes shall be connected. Notice that if *β* is large then it is very likely that two nodes in the grid may be connected. If $d(u,\,v)=\beta L$, then $P={e}^{-1}$, and $P < {e}^{-1}$ for every $d(u,\,v) > \beta L$. Thus $\beta L={d}_{p}$ has the significance of a *probabilistic* cut off distance, which determines with which probability $P$ nodes are joined, in contrast to the classical concept of deterministic cut off distance ${d}_{c}$, whereby after network conditioning, maximum edge length in the network is set as ${d}_{c}$.

Quantitative measure of the small-world-ness of a network is based on the knowledge of $Cc$ and $Cpl$ ^43^. A network $G$ with $n$ nodes and $m$ edges is a small-world network if it has a similar path length but greater clustering of nodes than an equivalent Erdos-Rényi (E–R) random graph with the same $m$ and $n$ (Supporting Information File 1). Let $Cp{l}_{rand}$ and $C{c}_{rand}$ be the mean shortest path length and the mean clustering coefficient for the E–R random graphs, and $Cp{l}_{graph}$ and $C{c}_{graph}$ the corresponding quantities for the graphs extracted from neural cells networks on rough silicon surfaces. If

2 $${\rm{\gamma }}=C{c}_{graph}/C{c}_{rand},{\rm{\phi }}=Cp{l}_{graph}/Cp{l}_{rand}\,$$

then the small-world-ness coefficient is defined as^43^

3 $${\rm{SW}}={\rm{\gamma }}/{\rm{\phi }}$$

The categorical definition of small-world network above implies ${\rm{\phi }}\ge 1$, ${\rm{\gamma }}\gg 1$, which, in turn, yields ${\rm{SW}} > 1$.

### Simulating information in cultured neural networks

We used a generalized leaky integrate and fire model^44, 45^ to simulate information flow in bi-dimensional neural networks as described in Reference^46^ and in separate Supporting Information File 3. Here, the nodes of the grid represent the nuclei of the cells and are extracted from confocal images of cultured neural networks at DIV 11. The temporal sequence of spikes that propagate along the grid encodes the information transmitted over that grid. Resulting patterns of multiple spike trains were interpreted using information theory approaches^47–49^. We represented the variability of individual neurons in response to a long random stimuli sample with the total entropy *H*. Similarly, the noise entropy *N* is the variability of the spike train in response to a sample of repeated stimuli. The information content provided by the different spike trains is the difference between entropies $I=H-N$.

### Functional multi-calcium imaging

Samples were incubated in 1 ml of dye solution (Fluo4, Life Technologies/Thermo Fisher diluted 1:2000 in Tyrode solution) at room temperature for 20 mins in dark conditions, washed once with Tyrode solution and immediately transferred to a recording chamber. Images were acquired with a Confocal upright microscope Leica SP5 AOBS; Leica Microsystems Srl) coupled with water-immersion objective lens (IRAPO 25X, 0.90 NA, Leica Microsystems Srl), and LAS AF software (Leica Microsystems Srl). Fluorophores were excited at 488 nm with an argon laser and visualized with a 510 nm–600 nm band pass emission filter. In each cell body, the fluorescence change Δ*F*/*F* was calculated as $({F}_{t}-{F}_{o})/{F}_{o}$, where ${F}_{t}$ is the fluorescence intensity at frame time $t$, and ${F}_{o}$ is baseline^31^. Spike timings were determined as the onsets of individual ${C}_{a}^{2+}$ transients. Calculation of calcium imaging signals from a region of interest was performed using the methods reported in ref. 50.

### Deriving the energy landscape of small world graphs

We generated bi-dimensional networks where the nodes of the network are sampled from normal distributions (Supporting Information File 4). On varying the number, position and standard deviations of the normal distributions over a significant range, we obtained networks with values of small-world-ness ranging from ~0.5 to ~3. Therefore, we associated an harmonic potential $e={k}_{s}\,{\delta }^{2}/2$ to each of the cell cell pairs in the ensemble. ${k}_{s}$ is the effective spring constant of the structural linkages between cells, *δ* is their separation. The potential describes the chemical interaction energy between cells due to specific (cell adhesion molecules, CAM, mediated adhesion) and not specific (electrostatics, electrodynamics, van der Waals) adhesion forces^41, 42, 51^. Summing the potential interaction energy over all the possible cell cell distances, we derived the total energy of cells clusters as a function of their degree of small-world-ness.

### Time evolution of cell density

The time evolution of neural cell density *u* in a mono-dimensional domain is described by the partial differential equation^38^

4 $$\frac{\partial u}{\partial t}=\frac{{\partial }^{2}u}{\partial {x}^{2}}-\frac{\partial \,u\,K(u)}{\partial x}+\gamma \,\eta (x){\alpha }_{2}(t){\rm{\Lambda }}(u)$$

where $x$ and $t$ are the space and time coordinates, $u\,K(u)$ is the cell-cell adhesive force proportional to $\xi$ (Supporting Information File 5). $\gamma \eta (x)\,{\alpha }_{2}(t)\,{\rm{\Lambda }}(u)$ represents the perturbation (instability) force that is related to surface roughness. In the term ${\rm{\Lambda }}$, it is lumped the dependence on the position on the substrate and cell density, ${\alpha }_{2}$ describes how rapidly the cell-substrate force component decays with time, $\eta$ reflects the random nature of the substrate, γ is the intensity of the substrate instability force. Thus ${\varrho }=\gamma /\xi$ is the relative intensity of the substrate perturbation force to the cell-cell adhesion force. Equation (4) was solved using a numerical scheme as described in a separate Supporting Information File 5.

### Data representation

We reported all averaged values as means ± standard deviations.

### Data availability

All data are available on contacting the corresponding author.

## Electronic supplementary material

Supporting Information File
