# Supplementary material for: Nano-topography Enhances Communication in Neural Cells Networks
Source: Sci Rep. 2017 Aug 29;7:9841. doi: 10.1038/s41598-017-09741-w (PMC5575309; doi:10.1038/s41598-017-09741-w)
Supplement: Supplementary file 1 — Supporting Information File [file 41598_2017_9741_MOESM1_ESM.pdf]

Nano-topography Enhances Communication in Neural Cells Networks

V. Onesto[a], L. Cancedda[b], M.L. Coluccio[a], M. Nanni[b], M. Pesce[b], N. Malara[a], M. Cesarelli[c], E. Di Fabrizio[a,d], F. Amato[a], F. Gentile[c,]*

[a] Department of Experimental and Clinical Medicine, University of Magna Graecia, 88100 Catanzaro, Italy
[b] Istituto Italiano di Tecnologia, Via Morego 30, 16163 Genova, Italy
[c] Department of Electrical Engineering and Information Technology, University of Naples, 80125, Naples, Italy
[d] King Abdullah University of Science and Technology, Thuwal 23955-6900, Kingdom of Saudi Arabia

*corresponding author: francesco.gentile2@unina.it

Supporting Information File

Index:

S.1 *Topological analysis of cultured neural cells networks* 2

S.2 *Wiring diagrams of cultured neural networks on rough surfaces* 5

S.3 *Simulating information in neural networks* 9

S.4 *Energy landscape of small world networks* 13

S.5 *Time evolution of cell density* 15

Supporting information file #1: Topological analysis of cultured neural cells networks

To have an indication of the connectivity properties of the nodes in a graph and to distinguish between graphs of different types (regular, random or small world), we quantified some network parameters including the clustering coefficient and the characteristic path length.

In graph theory, the clustering coefficient (C_c) is a measure of the degree to which nodes in a graph tend to cluster together. C_c ranges from 0 (none of the possible connections among the nodes are realized) to 1 (all possible connections are realized and nodes group together to form a single aggregate). The clustering coefficient is defined as

$$C_i = \frac{2E_i}{k(k-1)} \tag{S 1.1}$$

where k is the number of neighbors of a generic node i, E_i is the number of existing connections between those, $k(k-1)/2$ being the maximum number of connections, or combinations, that can exist among k nodes. Notice that the clustering coefficient C_i is defined locally, and a *global* value, C_c, is derived upon averaging C_i over all the nodes that compose the graph.

The characteristic path length (Cpl) is defined as the average number of steps along the shortest paths for all possible pairs of network nodes. We shall call the minimum distance between a generic couple of nodes the *shortest path length* (Spl), which is expressed as an integer number of steps.

Before computing the graph parameters, the connections between the nodes have to be established. A Waxman model[1,2] is used, whereby the probability of being a link between two nodes exponentially decreases with the Euclidean distance between those nodes. For a given set of two nodes *u* and *v*, the link probability, *P(u, v)* is defined as:

$$P(u,v) = \alpha e^{-d(u,v)/\beta L} \tag{S 1.2}$$

where *d* is the Euclidean distance between nodes *u* and *v*, and *L* is the largest possible Euclidean distance between two nodes of the grid. In the equation, α and β are the Waxman model parameters and, upon tuning these, the graph may be more or less dense. α and β should be chosen between 0 and 1. Selecting smaller values of these parameters results in a smaller number

of links. For the present configuration, these parameters were set to $\alpha = 1$ and $\beta = 0.025$. The probability P varies between 0 for a pair of nodes with an ideally infinite distance, and 1 for a pair of nodes with an ideally zero distance.

The information about the connections among the nodes in a graph is contained in the *adjacency matrix* $A = a_{ij}$, where the indices i and j run through the number of nodes n in the graph; $a_{ij} = 1$, if there exists a connection between i and j, $a_{ij} = 0$ otherwise. In the analysis, reciprocity between nodes is assumed, and thus if information can flow from i to j, it can reversely flow from j to i. In the framework of graph theory, we call a similar network an undirected graph. Notice that this property translates into symmetry of A being $a_{ij} = a_{ji}$. Moreover, $a_{ij} = 0$.

We showed above how to derive the distances between nodes d_{ij} in the networks. On the basis of d, we may decide whether a pair of nodes is connected, we use at this end the formula:

$$\alpha e^{-d_{i,j}/\beta L} - R \geq 0 \qquad \text{(S 1.3)}$$

in which R is a constant that we have chosen being 0.05 so that the probability of being a connection is $P = 0.95$.

With these premises, we show now how to calculate the Spl for a couple of nodes n_l and n_m.

In A, $a_{l,i}$ and $a_{i,m}$ account for all the pairs of nodes which are connected to n_l and n_m respectively. The sum of $a_{l,i}$ and $a_{i,m}$ over all the nodes in A, is stored in a new matrix $A_2 = \sum a_{l,i}\, a_{i,m}$ for all the l and m and A_2 has the same dimension of A. Now multiplicate A_2 and A repeatedly $A_2 = A_2\, A$, until all the terms of A_2 are non-zero and those terms in position ij will be the Spl between node i and node j. Finally, the characteristic path length Cpl is calculated like the average of Spl over A_2 [1].

Once obtained the C_c and cpl values, we defined a precise measure of ‘small-world-ness’, the ‘small-world-ness’ coefficient (SW), based on the trade off between high local clustering and short path length [3].

A network G with n nodes and m edges is a small-world network if it has a similar path length but greater clustering of nodes than an equivalent Erdos-Rényi (*E–R*) random graph with the same m and n (an *E–R* graph is constructed by uniquely assigning each edge to a node pair with uniform probability). Let Cpl_u and Cc_u be the mean shortest path length and the mean clustering coefficient for the *E–R* random graphs, obtained meaning the Cpl and the Cc of 20 uniform distributions, and Cpl_{graph} and Cc_{graph} the corresponding quantities for the graphs derived using the methods described above. We can calculate:

$$\gamma = \frac{Cc_{graph}}{Cc_u} \qquad \text{(S 1.4)}$$

$$\lambda = \frac{Cpl_{graph}}{Cpl_u} \qquad \text{(S 1.5)}$$

Thus, the 'small-worl-dness' coefficient is

$$\mathrm{SW} = \frac{\gamma}{\lambda} \qquad \text{(S 1.6)}$$

The categorical definition of small-world network above implies $\lambda \geq 1$, $\gamma \gg 1$ which, in turn, gives $\mathrm{SW} > 1$.

Supporting information file #2: Wiring diagrams of cultured neural networks on rough surfaces

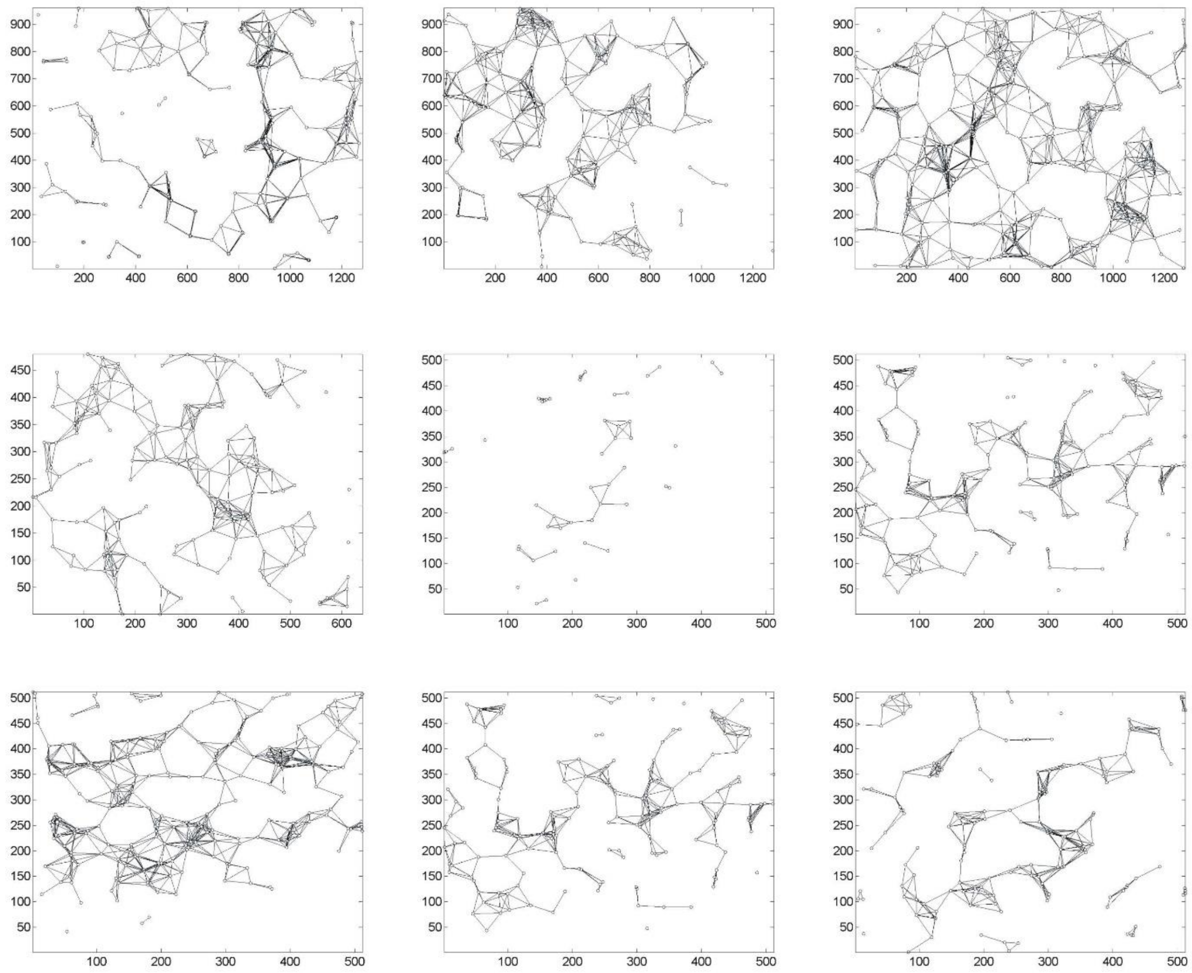

Supporting Information Figure 2.1: wiring diagrams of neural cells on substrate A

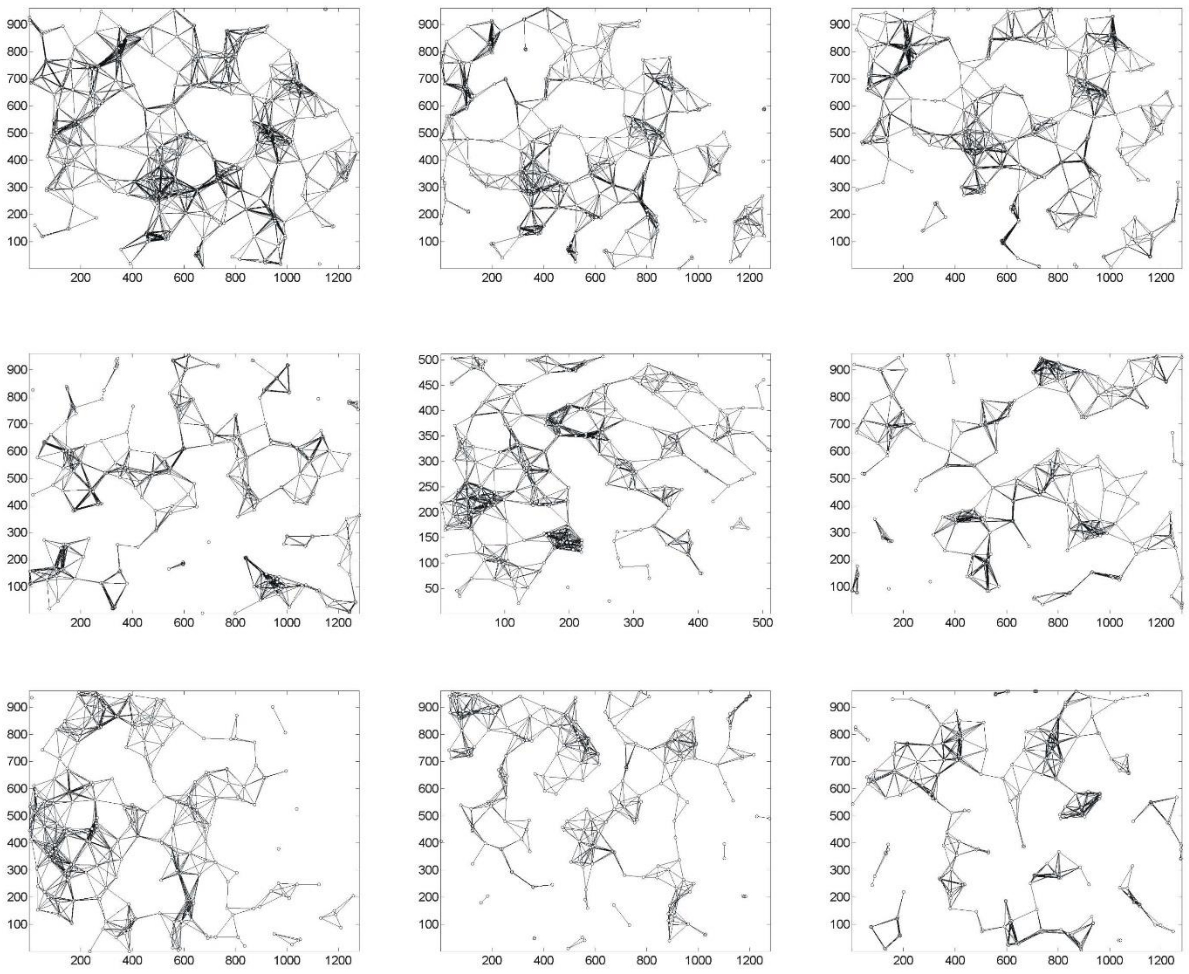

Supporting Information Figure 2.2: wiring diagrams of neural cells on substrate B

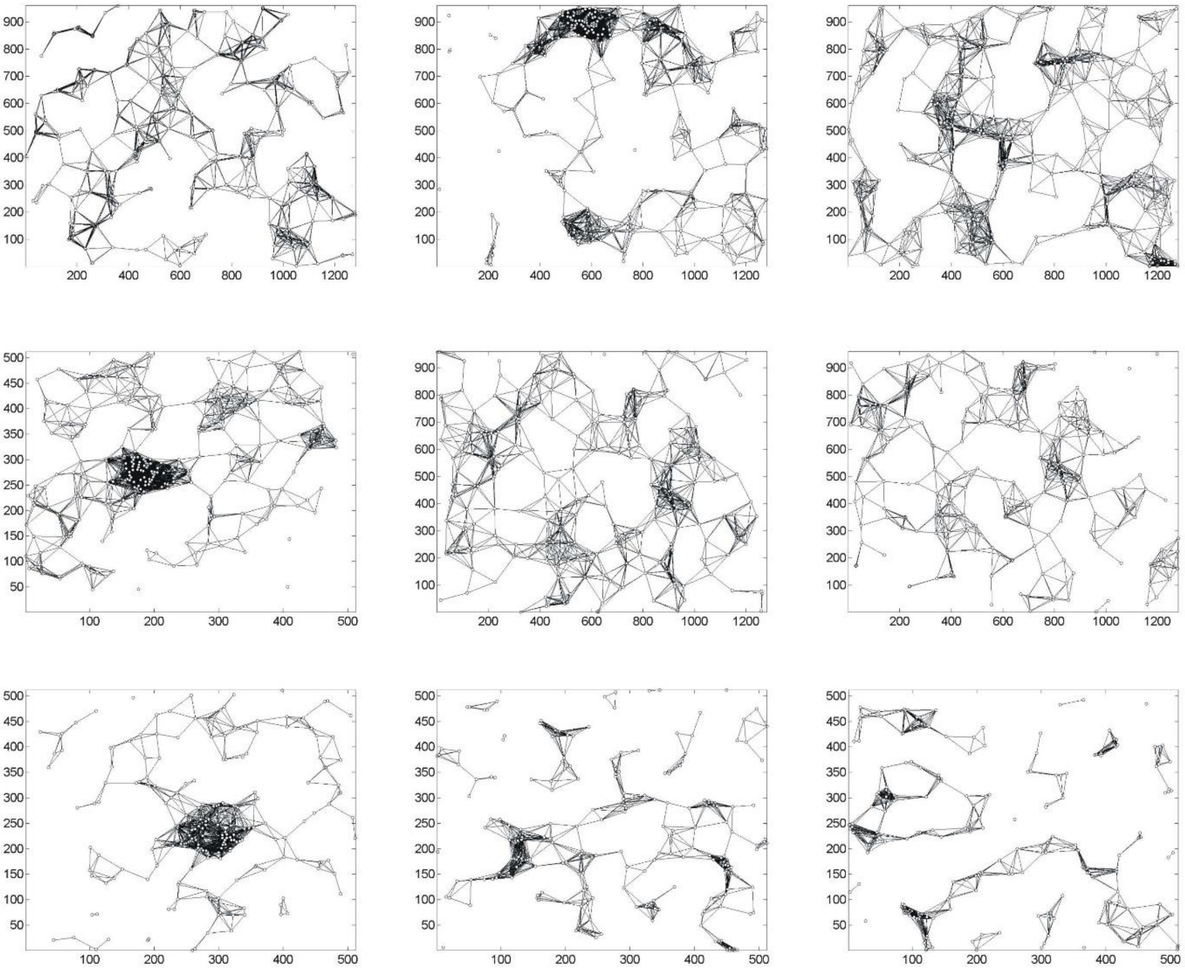

Supporting Information Figure 2.3: wiring diagrams of neural cells on substrate C

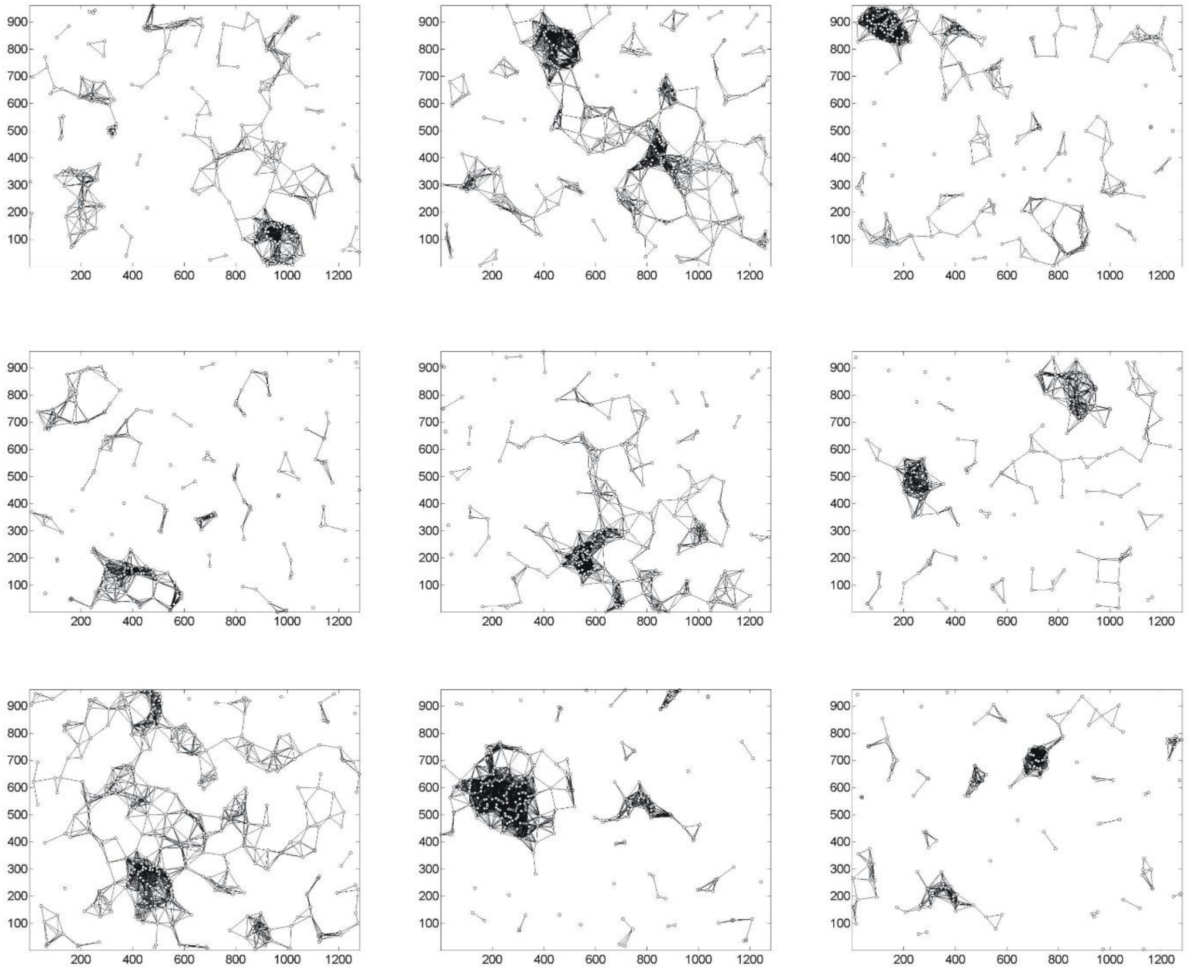

Supporting Information Figure 2.4: wiring diagrams of neural cells on substrate D

Supporting information file #3: Simulating information in neural networks

We used a generalized leaky integrate and fire model [4,5] to simulate trains of signals in bi-dimensional neural networks as described in Reference [6]. Here, the nodes of the grid represent the nuclei of the cells and are extracted from confocal images of cultured neural networks at DIV 11.

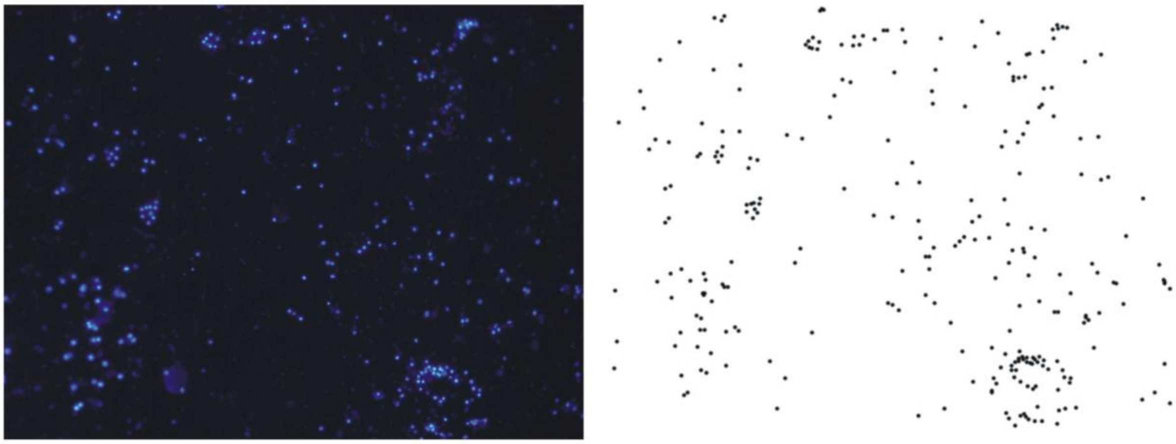

Supporting Information Figure 3.1

Nodes of the grid are therefore connected using the Waxman model as described in the methods of the paper and reference [1,7]. In doing so, we obtain undirected graphs where the edges of the graphs represent cell-cell connections (Supporting Information Figure 3.2).

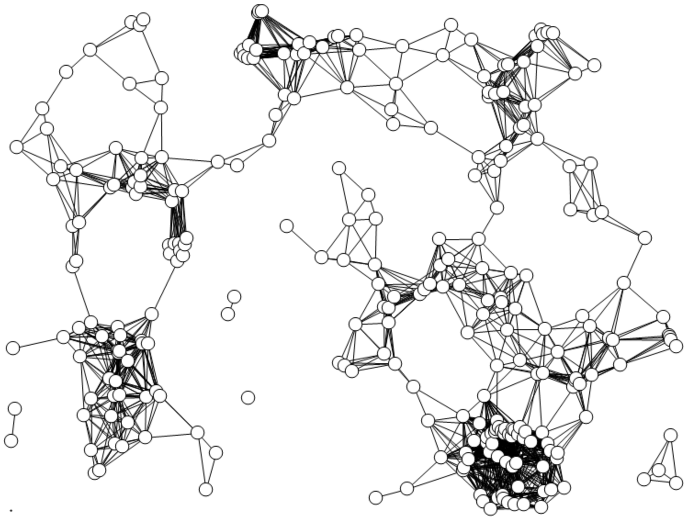

Supporting Information Figure 3.2

Then, nodes were randomly picked from the network and excited with a random (Supporting Information Figure 3.3a) and periodic (Supporting Information Figure 3.3b) signal of time. Upon excitation, spikes propagate in cascade in the grid.

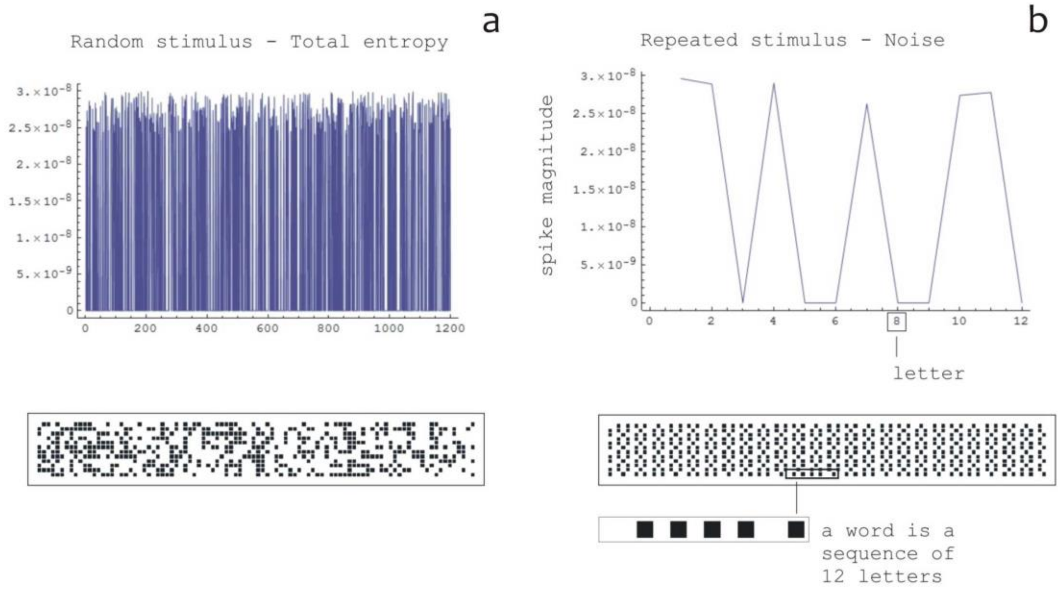

Supporting Information Figure 3.3

To analyse the flow of signal in the graphs, we used a generalized leaky integrate and fire model [4,5]. In individual neurons, electric pulses excite the neuron until the response (potential) at the postsynaptic sites reaches and surpasses a limiting value (that is, a threshold), then, the target neuron produces an impulse (an action potential) that propagates in turn to another neuron. This process is described by the following Equation, in which the membrane potential V obeys to a function of the sole time:

$$C_m \frac{dV}{dt} = -g_l(V - V_o) + I_{stim} \qquad \text{(S 3.1)}$$

Where C_m is the capacitance of the membrane, g_l is the conductance, V_o is the resting potential of the neuron. The current I_{stim} is the stimulus that excites the neuron until the membrane potential reaches a threshold V_{th} and an action potential is released from the system. Neurons in a grid are described by a set of coupled differential equations that generalizes the model described by Equation (S 3.1). Each node in the network sends and receives information and this process is mediated through the integrate and fire model and Equation (S 3.1). Assuming

linearity, I_{stim} is given by the superposition of current pulses J generated by the neurons i that fire on a neuron j

$$I_{stim}(t) = \sum_{i}^{\mathcal{B}} \zeta(d_{ij}) J \sum_{k}^{rel} \delta(t - t_i^k) \quad \text{(S 3.2)}$$

Where rel is the number of neurotransmitter release events, δ is the Dirac delta function, t_i^k is the timing of individual pulses. In Equation (S 3.2), ζ is a damping term which accounts for the inter-nodal distance d_{ij}. Pulses repeatedly excite a neuron until $V = V_{th}$ and an action potential is discharged from the target neuron. The action potential generates in turn an impulse that propagates through the network. The generation of an action potential at a node of the grid at a specific time is registered as an event (Supporting Information Figure 3.4). The temporal sequence of events encodes the information transmitted over that grid.

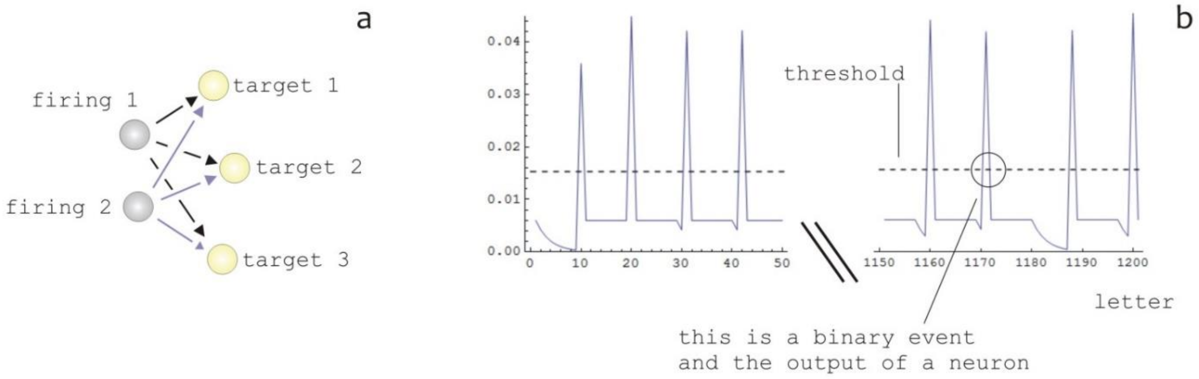

Supporting Information Figure 3.4

Resulting patterns of multiple spike trains were interpreted using information theory approaches [8-10]. Time spikes are grouped in sets of words, in which a word is an array of on (presence of a spike)/off (absence of a spike) events in a binary representation. On sorting words in order of decreasing occurrence in the train (Supporting Information Figure 3.5) we derived the associated Shannon entropy H using the following Equation:

$$H(S) = -\sum_{S} P(s) log_2 P(s) \quad \text{(S 3.3)}$$

that quantifies the average amount of information gained with each stimulus presentation. Entropy is measured in bits if the logarithm is taken with base 2. In the equation, $P(s)$ represents the probability with which a stimulus s is presented in the set S.

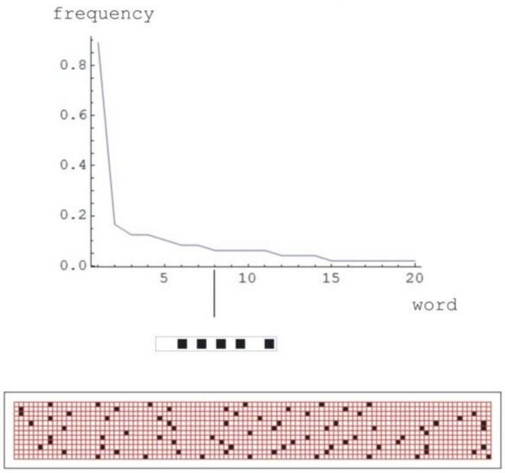

Supporting Information Figure 3.5

If H is the variability of individual neurons in response to a long random sample of stimuli (*total entropy*), and N is the variability of the spike train in response to a sample of repeated stimuli (*noise entropy*), then information that the spike train provide about the input is the difference between entropies $I = H - N$. This permits to derive information over all the nodes of the graph (Supporting Information Figure 3.6). In deriving information, we assume that networks are unidirectional. That is, given two adjacent nodes A and B in the grid (that is, directly connected by an edge), if information flows from node A to node B in the network, it cannot immediately afterwards flow from B to A. Thus the process of information propagation is irreversible and in general reciprocity does not hold in the grid. This accounts for the fact that, upon the generation of an action potential, a neuron experiences short-time synaptic depression and remains inactive for a refractory time τ. Thus, the travel of information in multiple directions is unrealistic in the short term and the present version of the model reflects this physical evidence. Notice though that, even if information cannot propagate from B to A in a direct passage and symmetry is broken, nevertheless patterns in the network may exist whereby information can re-circulate from B to A in complex paths. For this reason, the model can reproduce and simulate multiple events and spike trains in individual neurons.

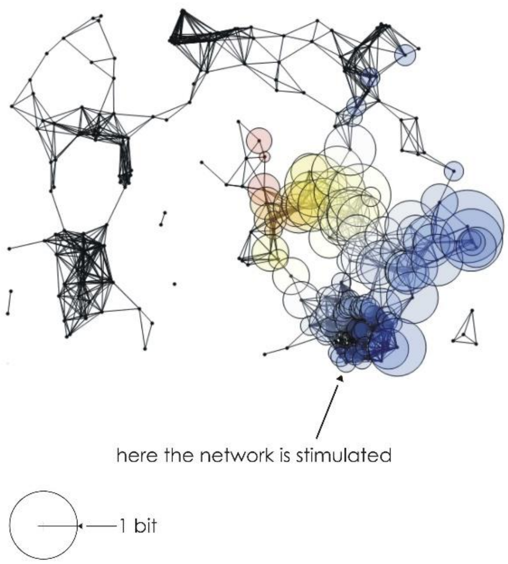

Supporting Information Figure 3.6

Supporting information file #4: Energy landscape of small world networks

We generated graphs having a number of nodes equal to $n = 150$ on squared grids (Supporting Information Figure 4.1); the nodes belong to a combination of five normal probability distributions, each one obtained using the *multivariate normal distribution* command in Matlab. These distributions have different configurations varying for mean (i.e. the distance between the sets of points) and covariance matrix (that is diagonal and contains variances along the diagonal and zero covariances off the diagonal). The variances values we used are (5, 10, 15, 20, 50, 100, 200, 500, 1000, 2000, 5000). For each combination of mean and variance, more than 100 configurations were generated.

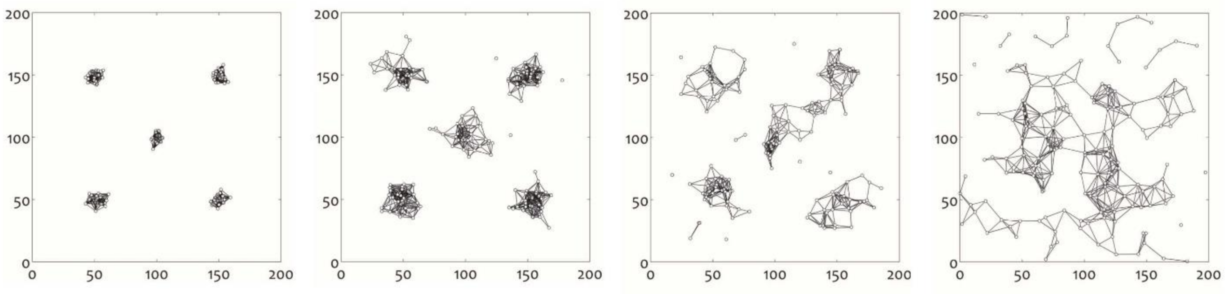

Supporting Information Figure 4.1

The connections between the nodes are obtained using the Waxman model described above (S1.2) with a probability of being a link between two generic nodes of $P = 0.95$ (S1.3). In doing so, we modulated network topology and thus networks small-world-ness between SW$\sim$0.6 and SW$\sim$3 (Supporting Information Table 4.1). Therefore, we associated an harmonic potential $e = k_s\,\delta^2/2$ to each of the cell cell pairs in the ensemble. Notice that, because we use the Waxman model, the number of connections is lower or equal to $n(n-1)/2$. k_s is the effective spring constant of the structural linkages between cells, δ is their separation. The potential describes the chemical energy of interaction between cells due to specific (cell adhesion molecules, CAM, mediated adhesion) and not specific (electrostatics, electrodynamics, van der Waals) adhesion forces [11-13]. Summing the potential energy of interaction over all the possible cell cell distances, we derived the total energy of cells clusters as a function of their degree of small-world-ness.

Variance	*Cc*	*Cpl*	*SW*
10	0.9817	1.0397	2.7060
100	0.7238	1.5913	1.2655
200	0.6153	1.6220	1.080
2000	0.5223	1.8138	0.8114
5000	0.4892	2.1241	0.654

Supporting Information Table 4.1

Supporting information file #5: Time evolution of cell density

The time evolution of neural cell density u in a mono-dimensional domain is described by the partial differential equation [14]

$$\frac{\partial u}{\partial t} = \frac{\partial^2 u}{\partial x^2} - \frac{\partial\, u\, K(u)}{\partial x} + \Upsilon\, \eta(x)\, \alpha_2(t)\, \Lambda(u) \qquad \text{(S 5.1)}$$

Where x and t are the space and time coordinates, $u\, K(u)$ is the cell-cell adhesive term and $K(u)$:

$$K(u) = \xi \int_{-1}^{+1} u(x+z)\omega(z)dz$$
$$\omega(z) = \begin{cases} -1 & -1 < z < 0 \\ +1 & 0 < z < 1 \end{cases} \qquad \text{(S 5.2)}$$

Thus cell-cell adhesion force is proportional to ξ. In Equation (S 4.1), $\Upsilon\, \eta(x)\, \alpha_2(t)\, \Lambda(u)$ represents the perturbation (instability) force that is related to surface roughness. In the term Λ, it is lumped the dependence on the position on the substrate and cell density:

$$\Lambda(u) = \int_{-1}^{+1} u(x+z)\omega(z)dz \qquad \text{(S 5.3)}$$

$\alpha_2(t) = e^{-\zeta\, t}$ describes how rapidly the cell-substrate force component decays with time, η reflects the random nature of the substrate, Υ is the intensity of the substrate instability force. Thus $\varrho = \Upsilon/\xi$ is the relative intensity of the substrate perturbation force to the cell-cell adhesion force.

We constructed a Finite Difference Scheme (FDS) in Matlab to find an approximate solution of the Partial Differential Equation S 5.1.

x is restricted to the finite interval $[p, q]$ of length $l_x = 20$, that is the *computational domain*. The dependent variable is $u = u(t, x)$.

The initial condition is

$$u(0, x) = r \qquad p \leq x \leq q \qquad \text{(S 5.4)}$$

The initial value of u for every x in the computational domain is a random generated value r from the uniform distribution on the interval $[a, b]$, where a and b are a down and up perturbation of 0.05 from a central value of 0.15.

Periodic boundary condition: we checked up when a solution variable has a position greater than a value inside the domain and subtracted the length of the domain from the position, reporting then that value to the position obtained.

Spatial and time discretization: the computational domain contains an infinite number of x values so first we must replace them by a finite set, by a grid of N equally spaced grid points. The constant grid spacing Δx is

$$\Delta \mathrm{x} = \frac{l_x}{m_x} \quad \text{(S 5.5)}$$

where m_x is the number of divisions that we have chosen equal to 100. Then $\Delta x \ = 0.2$. The spacing between time levels is $\Delta t = 0.02$. Fixing t at $t = t_n$, we approximate the spatial partial derivatives $u\_x = du/dx$ and $u\_xx = (d\^2\ u)/(dx\^2\ )$ at each point (t_n, x_i), using a *first order forward* difference scheme and a *second order symmetric* difference scheme respectively. Fixing x at $x = x_i$, we approximate the temporal partial derivative $u\_t = du/dt$ at each point (t_n, x_i), using a *first order forward* difference formula. So the final FDS is:

$$u_i^{n+1} = u_i^n + \frac{\Delta t}{\Delta x^2}\left(u_{i+1}^n - 2u_i^n + u_{i-1}^n\right) - \frac{\Delta t}{\Delta x}\left(u_{i+1}^n K(u_{i+1}^n) - u_i^n K(u_i^n)\right) + \Delta t\ \Upsilon\ \eta \alpha_2(n) \Lambda(u_i^n) \quad \text{(S 5.6)}$$

This is a *time-marching* scheme. Starting from the initial condition u_0, we used data for each grid point at time t_n to find data at each grid point at the future time $t_n + \Delta t$. This means that after an iteration of the scheme all u values at each grid point are known at time $t_n + \Delta t$. These new values are used as known data for another iteration of the scheme to give data for each grid point at the next time level. Solutions evolve from an initially random distribution and cluster together forming high density aggregates depending on the magnitude of ϱ.

References

1 Marinaro, G. *et al.* Networks of Neuroblastoma Cells on Porous Silicon Substrates Reveal a Small World Topology. *Integrative Biology* **7**, 184-197 (2015).

2 Waxman, B. Routing of multipoint connections. *IEEE Journal on Selected Areas in Communications* **6**, 1617–1622 (1988).

3 Humphries, M. D. & Gurney, K. Network 'Small-World-Ness': A Quantitative Method for Determining Canonical Network Equivalence. *PLoS ONE* **3**, e0002051 (2008).

4 de la Rocha, J. & Parga, N. Short-Term Synaptic Depression Causes a Non-Monotonic Response to Correlated Stimuli. *The Journal of Neuroscience* **25**, 8416–8431 (2005).

5 FitzHugh, R. Mathematical models of threshold phenomena in the nerve membrane. *Bulletin of Mathematical Biology* **17**, 257–278 (1955).

6 Onesto, V. *et al.* Information in a Network of Neuronal Cells: Effect of Cell Density and Short-Term Depression. *BioMed Research International* **2016**, 1-12 (2016).

7 Gentile, F. *et al.* Differential Cell Adhesion on Mesoporous Silicon Substrates. *ACS Applied Materials and Interfaces* **4**, 2903–2911 (2012).

8 Borst, A. & Theunissen, F. E. Information theory and neural coding. *Nature Neuroscience* **2**, 947 – 957 (1999).

9 Quiroga, R. Q. & Panzeri, S. Extracting information from neuronal populations: information theory and decoding approaches. *Nature Reviews Neuroscience* **10**, 173-185 (2009).

10 Strong, S. P., Koberle, R., van Steveninck, R. R. d. R. & Bialek, W. Entropy and Information in Neural Spike Trains. *Physical Review Letters* **80**, 197 (1998).

11 Bell, G. I. Models for the specific adhesion of cells to cells. *Science* **618**, 618-627 (1978).

12 Evans, E. A. & Calderwood, D. A. Forces and Bond Dynamics in Cell Adhesion. *Science* **316**, 1148-1153 (2007).

13 Sackmann, E. & Smith, A.-S. Physics of cell adhesion: some lessons from cell mimetic systems. *Soft Matter* **10**, 1644–1659 (2014).

14 Armstrong, N. J., Painter, K. J. & Sherratt, J. A. A continuum approach to modelling cell–cell adhesion. *Journal of Theoretical Biology* **243**, 98–113 (2006).
